# Responses of neurons in the marmoset primary auditory cortex to interaural level differences: comparison of pure tones and vocalizations

**DOI:** 10.3389/fnins.2015.00132

**Published:** 2015-04-20

**Authors:** Leo L. Lui, Yasamin Mokri, David H. Reser, Marcello G. P. Rosa, Ramesh Rajan

**Affiliations:** ^1^Department of Physiology, Monash UniversityClayton, VIC, Australia; ^2^Australian Research Council, Centre of Excellence for Integrative Brain Function, Monash UniversityClayton, VIC, Australia; ^3^Ear Sciences Institute of AustraliaSubiaco, WA, Australia

**Keywords:** primate, auditory cortex, response properties, sound localization, interaural level differences

## Abstract

Interaural level differences (ILDs) are the dominant cue for localizing the sources of high frequency sounds that differ in azimuth. Neurons in the primary auditory cortex (A1) respond differentially to ILDs of simple stimuli such as tones and noise bands, but the extent to which this applies to complex natural sounds, such as vocalizations, is not known. In sufentanil/N_2_O anesthetized marmosets, we compared the responses of 76 A1 neurons to three vocalizations (Ock, Tsik, and Twitter) and pure tones at cells' characteristic frequency. Each stimulus was presented with ILDs ranging from 20 dB favoring the contralateral ear to 20 dB favoring the ipsilateral ear to cover most of the frontal azimuthal space. The response to each stimulus was tested at three average binaural levels (ABLs). Most neurons were sensitive to ILDs of vocalizations and pure tones. For all stimuli, the majority of cells had monotonic ILD sensitivity functions favoring the contralateral ear, but we also observed ILD sensitivity functions that peaked near the midline and functions favoring the ipsilateral ear. Representation of ILD in A1 was better for pure tones and the Ock vocalization in comparison to the Tsik and Twitter calls; this was reflected by higher discrimination indices and greater modulation ranges. ILD sensitivity was heavily dependent on ABL: changes in ABL by ±20 dB SPL from the optimal level for ILD sensitivity led to significant decreases in ILD sensitivity for all stimuli, although ILD sensitivity to pure tones and Ock calls was most robust to such ABL changes. Our results demonstrate differences in ILD coding for pure tones and vocalizations, showing that ILD sensitivity in A1 to complex sounds cannot be simply extrapolated from that to pure tones. They also show A1 neurons do not show level-invariant representation of ILD, suggesting that such a representation of auditory space is likely to require population coding, and further processing at subsequent hierarchical stages.

## Introduction

The mammalian primary auditory cortex (A1) is critical for sound localization (Thompson and Cortez, 1983; Jenkins and Merzenich, 1984; Heffner and Heffner, 1990), and the responses of single A1 neurons encode information about sound source locations (e.g., Phillips and Irvine, 1981, 1983; Rajan et al., 1990; Recanzone et al., 2000; Mrsic-Flogel et al., 2005; Woods et al., 2006; Kusmierek and Rauscheker, 2014; see Grothe et al., 2010 for review). However, the spatial sensitivity of A1 neurons can vary with stimulus level (e.g., Brugge et al., 1996; Middlebrooks et al., 1998; Reale et al., 2003; Zhou and Wang, 2012). This is in contrast to psychophysical performance in both humans and monkeys, in which sound localization abilities remain relatively constant at intensities of 30 dB sound pressure level (SPL) and greater (Su and Recanzone, 2001; Recanzone and Beckerman, 2004; Vliegen and Van Opstal, 2004; Sabin et al., 2005). Pooling models where population responses are used to disambiguate sound location with changes in stimulus level can reconcile the discrepancy (Stecker et al., 2005; Miller and Recanzone, 2009). However, physiological studies to date have only used a limited range of auditory stimuli, primarily tones and/or noise bands, and thus we do not know whether such read-out strategies apply to more complex stimuli, including many types of natural sounds.

The social behavior of marmoset monkeys requires a range of context-specific vocalizations (Stevenson and Poole, 1976). These provide auditory stimuli that are both complex and biologically relevant (Miller et al., 2009), which have become the focus of many studies (i.e., Lu et al., 2001; Nagarajan et al., 2002; Eliades and Wang, 2003, 2013). Neurons in marmoset A1 responses reliabily to marmoset vocalization (i.e., Wang et al., 1995; Wang, 2000) including those which were anesthetized with sufentanil/N_2_O (Rajan et al., 2013). Although the spatial receptive fields in response noise-bands have been well characterized in marmoset A1 (Zhou and Wang, 2012, 2014), stimulus driven responses to complex stimuli may not necessarily reflect those elicited by noise bands and pure tones, given that nonlinear spectrotemporal interactions underlie A1 neuronal responses (Sadagopan and Wang, 2009).

The perception of a sound source derived from the processing of two types of binaural cues (interaural level differences [ILDs] and interaural timing differences [ITDs]), as well as monaural cross-frequency band comparison (Wightman and Kistler, 1997; Jin et al., 2004; Grothe et al., 2010). The latter predominates localization in the vertical plane (elevation), whereas localization in the horizontal plane (azimuth) depends on the two binaural cues. The range of ILD of the marmoset head-related transfer function is compatible with detection (Slee and Young, 2010); moreover, since ILDs are most useful for localizing high frequency sounds (Wise and Irvine, 1983; Grothe et al., 2010) and marmosets hears relatively high frequencies, ILD must be considered a strong cue for sound localization. Indeed, sensitivity to ILDs has been found in the nucleus of the brachium of the inferior colliculus of marmosets (Slee and Young, 2013), which is part of the input pathway to A1.

Here we address the extent to which A1 neurons code for ILDs in marmoset vocalizations as examples of complex naturalistic stimuli, and whether sensitivity to ILDs of vocalizations differ from sensitivity to ILDs in pure tones. Moreover, we test whether the neuronal encoding of ILDs changes at different overall SPLs. This study is directed to sound localization based specifically on ILD cues of which we hypothesize to be a major contributor. Our data yield new information regarding primate A1 responses to behaviorally relevant complex sounds, which can facilitate the development of models of sound localization based upon ILD in natural conditions.

## Experimental procedures

Experiments were conducted in six adult marmosets (*Callithrix jacchus*), in acute (24–72 h) recording sessions that targeted the auditory cortical areas on the surface of the left superior temporal gyrus. Experiments were approved by the Monash University Animal Experimentation Ethics Committee, which also monitored the welfare of the animals. All procedures followed the guidelines of the Australian Code of Practice for the Care and Use of Animals for Scientific Purposes.

### Preparation

The preparation has been described in detail previously (Rajan et al., 2013) and closely followed the protocols used in previous studies of visual (e.g., Lui et al., 2006) and motor (Burman et al., 2008) cortex physiology in marmosets. Each animal was pre-medicated with intramuscular injections of diazepam (3 mg/kg) and atropine sulfate (0.2 mg/kg). After 20 min, anesthesia was induced with intramuscular alfaxalone (Alfaxan, 10 mg/kg; Jurox, Rutherford, Australia), allowing tracheotomy, vein cannulation and craniotomy to be performed. After all surgical procedures were completed, the animal was administered an intravenous infusion of sufentanil (8 μg/kg/h; Janssen-Cilag, Sydney, Australia) and dexamethasone (0.4 mg/kg/h; David Bull, Melbourne, Australia) diluted in lactated Ringer's solution (injection volume 1.5 ml/h). Artificial ventilation with a gaseous mixture of nitrous oxide and oxygen (7:3) was delivered via the tracheal cannula. The electrocardiogram, blood pressure and SpO_2_ level were continuously monitored, and core body temperature was maintained at 38 ± 0.5°C using a thermostatically controlled electric blanket regulated by a rectal thermometer. A head bar held in a stand was fixed to the forehead using a short screw and dental cement to hold the head rigidly. The ear canals were surgically exposed to allow the insertion of sound delivery tubes connected to Sennheiser headphone speakers (Rajan, 2000). This preparation allowed very stable recordings, including monitoring of the same cells over periods in excess of 2 h (Bourne and Rosa, 2003; Rajan et al., 2013). Targeting of the electrode toward A1 was based on previously published maps (Aitkin et al., 1986; de la Mothe et al., 2006; Reser et al., 2009; Paxinos et al., 2012) and direct visualization of the lateral sulcus, which was visible through the silicone oil-covered dura mater.

### Stimuli

Presentation of stimuli, together with acquisition and processing of neural responses, was performed using custom-developed Matlab software (MathWorks, Natick, MA), which has been employed in previously published studies (Rajan et al., 2013). Sounds were delivered using probe tubes fitted snugly into the external ear canal, at a distance of about 1 mm from the eardrum. SPL was calibrated using a type 2673 microphone, powered by a type 2804 microphone power supply, against the speaker output probe tube, and feeding into a Bruel and Kjaer (Copenhagen, Denmark) sound level calibrator type 4230 (94 dB @ 1000 Hz).

Pure tone stimuli (100 ms duration) were generated using TDT hardware (Tucker Davis Technologies, Alachua, FL). Tones were created in a TDT RX6 multi-function processor (which also controlled the presentation of vocalization stimuli, see below). Stimuli were filtered using a low-order 10 kHz highpass filter to flatten the speaker output (which was otherwise strongly biased to low frequencies): The output of the speakers was such that the unfiltered output was 20–30 dB greater from 1 to 3 kHz than at frequencies from 8 to 40 kHz, where the output was flat within ± 3 dB. This would create problems in trying to generate a broad band stimulus where the output at individual frequency components was needed to be equal. To overcome this issue, a 10 kHz high pass filter was applied, so that the output at lower frequencies was attenuated to “flatten out” the maximum output across frequencies at the speaker was very similar. The high-pass filter had a slope of 10 dB/octave, reducing the speaker output at the lower frequencies to levels similar to those from 8 to 40 kHz. Note that this has no effect on any across-frequency variations in level within a call (see below), but allowed the output for a broad band noise stimulus to be even across-frequency. This was then passed to a PA5 programmable attenuator and subsequently to an HB7 headphone driver (Tucker Davis Technologies), before delivery via the speakers.

**Figure 1:**
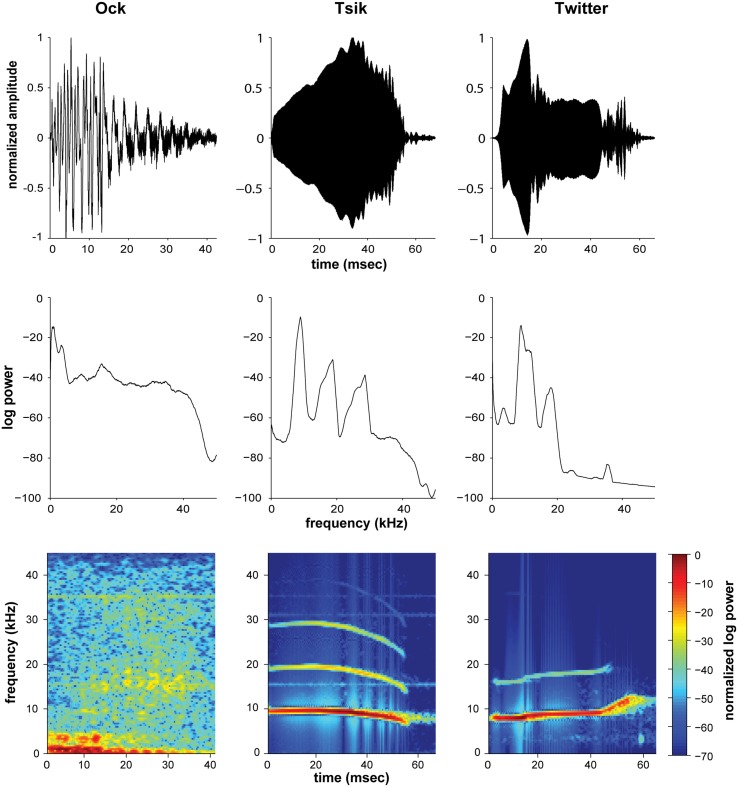
**Waveforms (top), Fourier transforms (middle) and spectrograms (bottom) of the three natural stimuli used in this study, the Ock (left), Tsik (center) and the first syllable of Twitter (right)**. The Fourier transforms and spectrograms were computed using hamming windows of length 512 data points, with overlaps of 500 samples. Values shown are the logarithm of the mean power across windows, normalized with respect to maximum power as indicated by color bar for the spectrogram.

Three recorded marmoset calls (“Ock,” “Tsik,” and “Twitter”; Epple, 1968; Aitkin and Park, 1993; Figure 1) were also used for study. These calls served as examples of complex, biologically relevant auditory stimuli. Only one call (token) per each type of vocalization was used per experiment to ensure that neural variability was not due to the variation in stimulus. In behaving marmosets, “Ock” and “Tsik” are social mobbing calls, used to attract and potentially provide cues necessary for localization by conspecifics, whereas “Twitter” is a contact call, presented upon visual contact with another conspecific (Aitkin and Park, 1993). Comparatively, the Ock call is a broadband stimulus, not only with power in the lower frequencies, but also with power distributed through the entire audible range of the marmoset. Meanwhile, the Tsik and Twitter have similar frequency characteristics, carrying multiple narrower bands of spectral energy from around 7 kHz up to 20 and 30 kHz for the Twitter and Tsik, respectively (see Figure 1).

Calls were recorded from monkeys not included in this sample, using Aco Pacific Type 1 microphones (Model 7012, Aco Pacific, Belmont, CA) in a sound-attenuated chamber. Microphone output was acquired via a preamplifier by a Tascam HD-P2 digital recorder (192 kHz A/D rate; TEAC America, Inc., Montibello, CA) and stored as individual.WAV files. Specific calls were extracted from the recording using a custom MATLAB interface, which allowed an experienced user to classify each call type and capture it as a separate file. Call files were trimmed, normalized in amplitude, and played at the same rate of 192 kHz. For calibration, the.WAV file was played out at maximum amplitude by the TDT system, through the same sound delivery speaker used in experiments, into a closed coupler system that contained the condenser microphone placed against the sound delivery tube. The condenser microphone sensitivity function was used to calibrate the system output as the SPL averaged over the call duration. This was used to generate.WAV files of recorded calls at other desired SPLs.

### Testing for ILD sensitivity

Neuronal sensitivity to ILDs was tested for each stimulus. ILD sensitivity was tested using the Average Binaural Level (ABL) constant method, in which ILDs are generated by varying the level of the stimulus in two ears symmetrically around a base (average) binaural level (Irvine, 1987). For each stimulus three ABLs were used: 30, 50, and 70 dB SPL, and for each ABL, nine ILDs were generated by varying the levels in the two ears symmetrically around that ABL. Test ILDs used for each stimulus ranged from −20 dB (20 dB louder in the ear contralateral to the recording A1) to +20 dB (20 dB louder in the ear ipsilateral to the recording A1), usually in 5 dB increments, and always included 0 (same level in both ears). This stimulus set encompasses the majority of the range of ILDs measured in the marmoset, for frequencies that are predominate in the vocalizations we used (Slee and Young, 2010; Rajan et al., 2013).

### Recordings

All recordings were done with the animal inside a sound-attenuated and lightproof room. Parylene-coated tungsten electrodes (FHC, Bowdoin, ME) with an impedance of 2–4 MΩ at 1 kHz were positioned approximately normal to the cortical surface. The electrodes were advanced through the intact dura mater until the first depth at which responses from a multi-unit cluster could be reliably observed above background activity (generally when the response amplitude was about 1.5 × mean noise level). The dura mater (covered in silicone oil) was readily penetrated by the electrode, and first recordings were generally obtained within 100–200 μm of the surface. From this point, multi-unit clusters were sampled at approximately 150 μm intervals, until the white matter was reached (approximately 2–3 mm from the pial surface, depending on the electrode's angle relative to the banks of the lateral sulcus). As expected, neurons recorded at adjacent sites had similar characteristic frequencies (CFs). Nonetheless, other response properties changed, confirming that the separations between recording sites were sufficient to avoid repeated recordings from the same cell. Sampling (25 kHz), amplification (×1000) and filtering (bandpass 500 Hz–5 kHz) of the electrophysiological signal were achieved by a Model RA4PA Medusa Pre-amplifier and an RA16 Medusa Base Station (Tucker Davis Technologies). Data were stored digitally for both online and offline processing.

At all recording sites, the CF and threshold of the multi-unit responses were first determined under interactive control by the experimenter, using online monitoring of neuronal responses while pure tone stimuli were varied in frequency and level (Rajan et al., 2013). This assessment was followed by a systematic characterization of the responses to a randomized array of stimuli consisting of up to 22 pure tones with frequencies linearly spaced from 500 Hz to 32 kHz, and up to eight amplitudes (10–80 dB). Each tone (100 ms duration, 0.5 ms cosine rise–fall ramps) was presented binaurally at equal levels, with a 500 ms inter-stimulus interval. The full stimulus matrix was presented a minimum of 10 times, and the responses to each frequency–level combination were summed online and displayed as tone frequency–level response areas (Rajan, 1998; Carrasco and Lomber, 2009). The CF was then determined as the frequency at which responses could be reliably evoked at the lowest intensity in the frequency–level response areas.

**Figure 2:**
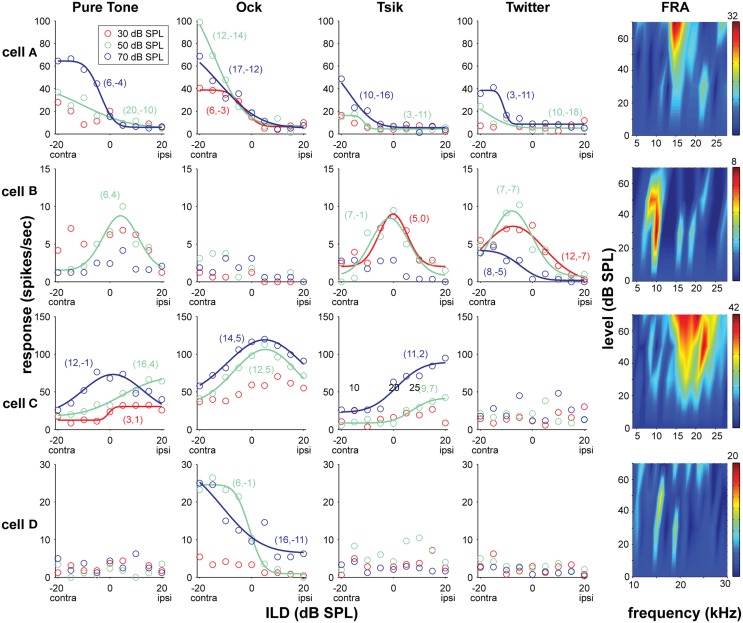
**Examples of four A1 neurons' responses to ILD for different mean intensities (ABL) and stimulus types; the frequency-level response areas (FRA) are also shown**. Data from each cells **(A–D)** occupy the same row, and the response to the different stimulus types (pure tone, Ock, Tsik, Twitter, and FRA) are arranged in columns. The color code identifies the ABL as per the legend in the top-left sub-plot. For any stimulus/ABL combinations that were sensitive to changes in ILD (*p* < 0.005), the optimal model (solid line) is also illustrated. Negative ILD values refer to ILDs that are louder in the contralateral (contra) ear, and positive values refer to ones louder in the ipsilateral (ipsi) as indicated below the values on the x-axis. The values in the brackets, show in that order, are the fitted σ values and the midpoint (for monotonically selective responses) or peak (for peak selective cells) as indicated by the fitted value d_0_. The colors of these values correspond to their respective ABLs as indicated by legend. CFs for cell **(A)** 15 kHz, **(B)** 10 kHz, **(C)** 22 kHz, **(D)** difficult to define, recorded at the area of ≈28 kHz which was used. Spike rates in the FRAs are indicated by the color bar on the very right, the number on the top indicates the maximum value in spikes/second.

After CF determination, sensitivity to ILDs was tested using a pure tone of appropriate CF for the cell, as well as the three vocalizations. Each of these stimuli was presented at 3 ABLs and 9 ILDs. The order of test conditions was randomized, and at least 20 trials were conducted in each test condition; for example, each panel in Figure 2 contains data from at least 540 trials (3 ABL × 9 ILD × 20 trials).

### Data analysis

Single and multi-unit data were extracted from the recorded signal using the Offline Sorter program (Plexon Inc., Dallas, Tx). Single units were identified by separation of spike waveform and principal components analysis. Typically only one type (single or multi) of neural recording was extracted from each recording site. For analysis both single and multi-units were analyzed and the results presented in this report consist of data from both types of neural recordings presented together. Spike time data were exported from the Offline Sorter to MATLAB, and all subsequent analyses were performed using purpose-written MATLAB programs.

A single trial response was computed as the mean firing rate over the first 100 ms after stimulus onset. The 100 ms matched the duration of the pure tone; while the durations of all calls were shorter than 100 ms, the neural responses to the calls generally lasted for at least 100 ms and therefore we opted to keep the analysis time window consistent between stimuli. The 100 ms interval represents a reasonable time for neural activity to be read-out for a behavioral response, and a similar window has been used in other studies of sound localization encoding in marmoset A1 (Zhou and Wang, 2012).

The level of spontaneous activity was defined as the mean firing rate in the 50 ms prior to stimulus onset. Only cells that responded at a level at least two standard deviations above the mean spontaneous rate at the optimal average binaural level (ABL_opt_) and ILD for at least 1 call or the pure tone were included in subsequent analyses. To determine if an individual cell exhibited ILD sensitivity for a particular stimulus across all ABLs, we applied a Two-Way ANOVA, with the independent variables being ILD and the ABL level (SPL) in that test condition. A cell was considered to be sensitive for ILD if there was a significant main effect for ILD, and/or a significant interaction effect of ILD and ABL on the firing rate. To correct for the fact that we were conducting this analysis four times for each cell, once for each stimulus type, we applied a Bonferroni correction to choose the conservative value of α = 0.01.

To assess whether cells were sensitive to ILD at a given ABL for a particular stimulus, we used a One-Way ANOVA (*p* < 0.005); again, this conservative value was adopted to correct for multiple comparisons, as we were conducting this analysis three times (three ABL levels) for each stimulus, equating to 12 times per unit (4 stimuli × 3 analyses). In addition, we also determined ILD sensitivity type and parameters for all significant cells by fitting two ILD-response functions below, a monotonic (Equation 1) or peaked (Equation 2) function, consistent with the large body of literature segregating ILD functions in auditory cortex and lower levels of the auditory pathway into these two predominant classes. The sensitivity type was assigned on the basis of the best fitting function as described below.

Equation (1) is a sigmoid, representing a monotonic relationship between the ILD and cell response as measured by spike rate:

(1) $$\text{R}(\text{d})=\text{B}+\text{A erf }[(\text{d}-{\text{d}}_{0})/\sigma ](\text{erf is an error function})$$

Here, R(d) is the fitted responses with respect to d, where d is the ILD. The four free parameters are A, which represents the peak firing rate with the baseline response subtracted, d_0_, representing the midpoint of the sigmoid function, σ, representing the slope of the ILD-response, and B representing the baseline response.

For peaked ILD functions, we fitted the following Gaussian function:

(2) $$\text{R}(\text{d})=\text{B}+\text{A exp}[-0.5({\left(\text{d}-{\text{d}}_{0}\right)}^{2}/\sigma^{2})]$$

where R(d) represents the fitted response with respect to ILD, and d is ILD. Again, this function has four parameters: A again represents the amplitude (maximum—baseline), d_0_ the position of the peak representing the optimal ILD, σ gives the slope, and B is the baseline response. In both instances, parameters were optimized using the “lsqcurvefit” function in MATLAB. The amplitude of the functions (parameter A) was constrained for both Equations (1) and (2), so that it never exceeded 1.2 times the modulation range (maximum response minus minimum response) of the cell; this minimized the probability of artificial peaks being fitted to the data, as confirmed by visual inspection of the curves. The σ is a measure of relative slope with respect to the amplitude (parameter A); large σ values would indicate that the rate of change in spiking activity that accompanied changes in ILD occurs relatively slowly, conversely, a smaller σ would indicate the rate of change is more abrupt. For the Gaussian function, this parameter which can be measured in this case in dB of ILD is usually thought of as a measure of the width of the bell curve, a measure directly dependent on the slope, and hence the σ parameter can also to be a measure of slope. In terms of the absolute change in firing rate with respect to change in ILD, this is clearly highly non-linear and depends on other parameters of the model and also the ILD in question.

As both functions had four free parameters, the optimal model was determined by comparing coefficients of determination (*R*^2^) calculated using the residual to each fit. Responses that had higher *R*^2^ values to the Gaussian function were considered to exhibit peaked sensitivity, whereas responses that had higher *R*^2^ values to the sigmoid function were sensitive to ILD in a monotonic manner. In our convention, monotonic cells with parameter *A* < 0 had a higher firing rate to the contralateral side for a given stimulus/ILD combination (negative ILD values denoting louder in the contralateral ear), and *A* > 0 indicates cells that preferred the ipsilateral side.

To determine a neuron's optimal ABL for ILD sensitivity, we calculated the following *discrimination index* (DI):

(3) $$\text{DI}=\frac{{\text{R}}_{\text{max}}\;-\;{\text{R}}_{\text{min}}}{R_{\text{max}}-R_{\text{min}}\;+\;2\sqrt{\text{SSE/}(\text{N}-\text{M})}}$$

where R_max_ and R_min_ are the maximum and minimum firing rates respectively, SSE is the sum squared error around the mean response, N is the number of observations, and M is the number of stimulus values tested. This index was chosen as it takes into account neuronal variability, in addition to the means. This index has the advantage of considering the ability of a neuron to discriminate changes in the stimulus relative to its intrinsic level of variability (Prince et al., 2002; DeAngelis and Uka, 2003). We also calculated the *modulation range* of the firing rate as R_max_−R_min_ which does not take into account the variance of responses.

We used *t*-tests to compare means. Either parametric (ANOVA and *t*-test) or non-parametric (Kruskal-Wallis, Wilcoxon rank-sum test) measures were used to compare means/medians depending on normality of distributions; these are identified where applicable in the reporting of the results.

### Histology

At the end of the experiment the animal was administered an overdose of sodium pentobarbitone (300 mg/kg) (Rhone-Merieux, Brisbane, Australia) and perfused transcardially with 0.9% saline, followed by 4% paraformaldehyde in 0.1 m phosphate buffer (pH 7.4). After cryoprotection in increasing concentrations of sucrose (10–30%), and sectioning (40 μm, coronal plane), alternate sections were stained for Nissl substance, myelin (Gallyas, 1979) and cytochrome oxidase (Wong-Riley, 1979), for the reconstruction of electrode tracks relative to histological borders (see Rajan et al., 2013). Electrode tracks were reconstructed with the aid of small electrolytic lesions (4μA, 10 s), which were placed at various sites during the experiments. The laminar distribution of the recorded units was also determined based on the cytoarchitecture.

Determination of the identity of the areas from which recordings were obtained used criteria defined by previous studies (de la Mothe et al., 2006; Reser et al., 2009), and the recent stereotaxic atlas of the marmoset brain (Paxinos et al., 2012). One of the principal histological features that characterizes A1 is a thick band of dense cytochrome oxidase reactivity in the middle cortical layers (including the lower part of layer 3, but centered on layer 4). The characteristics of this cytochrome oxidase band allow discrimination of the A1 from the adjacent core and belt areas. The myelination pattern of A1 relative to the latter areas is another useful indicator of the anatomical boundary (de la Mothe et al., 2006).

## Results

The responses of 76 A1 units from six animals, to CF pure tones and three natural marmoset vocalizations (Ock, Twitter, and Tsik), were characterized, using ILDs from −20 dB to +20 dB created at three ABLs. The majority of these recordings (66%) were multi-units. All recording sites in the present report were confirmed to be in A1 by histological reconstruction of the electrode tracks, which revealed that the sample included cells from layers 2 to 6. The CFs ranged from 0.9 to 31 kHz, with a median of 12 kHz. 75% of these cells had a CF between 5 and 20 kHz, a range that covers most of the energy content of the vocalizations tested (see Figure 1). Only 12 cells had CFs ≥ 20 kHz.

The total number of cells presented with each stimulus is shown in Table 1; of the 76 cells, the majority of these (62) were tested with all four stimuli, 10 were tested with three out of the four stimuli, and the remaining four cells had data from two stimuli. The number of cells sensitive to ILD is also shown in Table 1, and this depended on stimulus type [χ^2^_(3)_ = 9.23, *P* = 0.026]: more cells were sensitive to the Ock (77%) and the appropriate CF tone (78%) compared to the number of cells sensitive to the Tsik (63%) and Twitter (69%). The fact that large numbers of cells were responsive for any given call may be explained by that fact that these calls have spectral power across a broad range of frequencies, including the plateau spectrum range outside the main spectral peak, and would therefore cover at least some part of the response area of these cells. Five (of the 76) cells did not respond differentially to ILDs in any of the four stimuli. These cells were found adjacent to cells which did respond to ILDs in two or more of the stimuli, suggesting that the lack of sensitivity reflected a genuine neuronal property, and not inadequate stimulation of the cell by the acoustic stimuli (see Discussion).

**Table 1:** **The number of cells that were tested for each stimulus, the number that were ILD sensitive and which ABI these preferred**.

	**Total cells**	**ILD sensitive**	**ABL_opt_ 30 dB**	**ABL_opt_ 50 dB**	**ABL_opt_ 70 dB**
CF pure tone	67	52	19	13	20
Ock	73	58	10	24	24
Tsik	75	46	4	9	33
Twitter	71	42	7	5	30

### Types of sensitivity functions for ILD

**Figure 3:**
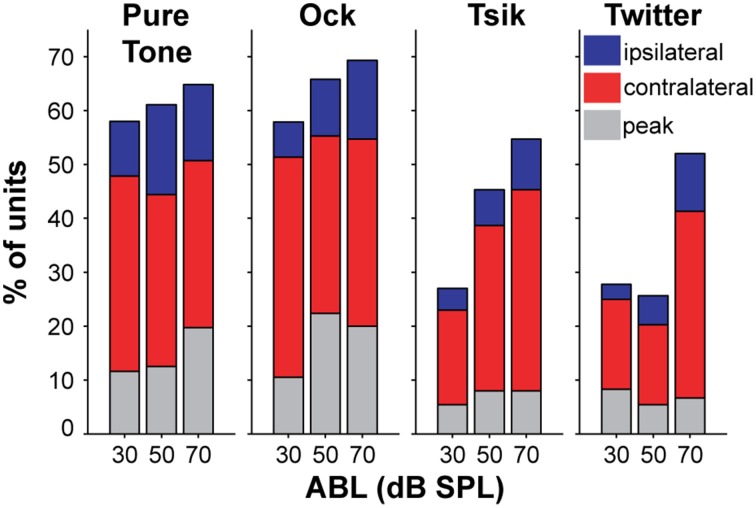
**Percentage of sensitivity type of for each stimulus type at each ABL**. Each sub-plot shows data for each stimulus type, and each bar provides data for each ABL. Color code as illustrated by the legend on the right sub-plot indicates the type of ILD-sensitivity function.

Figure 2 shows the response patterns of four cells which exemplify the types of ILD sensitivity observed in A1. Cell A was always monotonically sensitive, favoring the contralateral ear regardless of stimulus type; this was the most frequently observed pattern in A1, representing approximately 59% of the total sample (Figure 3: red bars). Cell B is an example of a cell with sensitivity that, when present, is generally best described by a peaked function (here for pure tone, Tsik, and Twitter stimuli). Cell C's sensitivity to ILD depended on both stimulus type and ABL. Both peaked and monotonic responses were observed, depending on the stimulus and ABL (e.g., pure tone response). Interestingly, this cell was sensitive for ILDs in the Tsik call, but did not respond to the Twitter call, which has a similar frequency spectrum. The activity of Cell D can also be considered as complex, as its responses to certain stimuli were not predictable based on their spectral content; it responded only to the Ock stimulus, a stimulus which encompasses this cell's CF of 26 kHz, yet it did not show any response to ILDs in CF pure tones, or other calls.

It is also worth noting that where cells were deemed to be insensitive to ILD for a particular stimulus/ABL combination, in the majority of cases (87%) these cells were actually non-responsive to that combination (e.g., Cell D, Twitter responses, in Figure 2). Conversely, where cells were responsive to a given stimulus/ABL combination, the majority (78%) of these responses were significantly sensitive to ILD (*p* < 0.005), consistent with the fact that ILD is a major stimulus parameter influencing A1 neurons.

**Figure 4:**
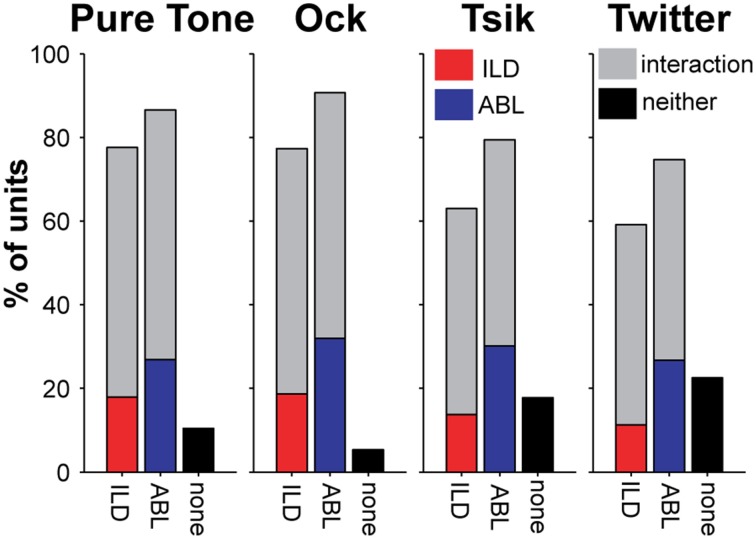
**Percentage of cells sensitive to ILD and ABL for each stimulus type**. The total percentages of cells sensitive to ILD and to ABL (including interaction effect) for each stimulus are shown in the four sub-plots. The color code indicates whether cells are sensitive to only one variable, or have a significant interaction effect. Cells are considered to be sensitive to ILD if there is a significant main effect for ILD and/or a significant interaction of ILD and ABL (Two-Way ANOVA, *p* < 0.01). The corresponding rule applies to ABL. These categories are not mutually exclusive, i.e., a cell can be sensitive to both ILD and ABL and therefore would contribute to both bars; however, each cell can only contribute to a single bar only once, i.e., cells with interaction effects will appear in gray regardless of main effect(s).

As demonstrated by the examples shown in Figure 2, the pattern of ILD sensitivity for each stimulus type almost always depended on ABL. This is summarized for our population of cells in Figure 4; each subpanel presents data for one stimulus type. For all stimuli, the majority of cells were sensitive to ILD (first bar in each subpanel). However, the proportion of ILD-sensitive units was always marginally lower than that of cells sensitive to ABL (second bar in each subpanel), and more importantly, the majority of ILD-sensitive cells showed a significant interaction with ABL (the gray segment of each bar in these subpanels) irrespective of stimulus. This is exemplified by Cell C in Figure 2: its sensitivity (or the lack thereof) always depended on the ABL for every stimulus to which it was responsive. Cells that showed an interaction effect represented a large majority of the total cells tuned for ILD (76–81%); these percentages were not different between stimuli [χ^2^_(3)_ = 0.02, *p* > 0.05; Figure 4]. The percentage of cells that showed an interaction between ILD and ABL was about 50–60% across all stimuli (gray bars in Figure 4), and this difference corresponded to the total number of cells that were sensitive to ILD.

The distribution of the different patterns of ILD sensitivity for each of the four stimulus types is illustrated in Figure 3, with data segregated according to ABL. For the ILD-sensitive cells, the most common response function was monotonic (blue + red segments of bars in Figure 3; e.g., Figure 1, Cell A, for most ABLs and all stimuli), with 59% of ILD-sensitive cells favoring the contralateral ear (red segment of bars in Figure 3; e.g., Figure 2, Cell A), and 18% of ILD sensitive cells favoring the ipsilateral ear (blue segment of bars in Figure 4; e.g., Figure 2 Cell C, Tsik and CF tone stimuli). The remaining 23% of ILD-sensitive cells were best described by a peaked function (gray segment of bars in Figure 3; e.g., Figure 2, Cell B, Tsik and Twitter calls). The distribution of sensitivities did not differ across stimulus type or level [χ^2^_(11)_ = 19.7, *p* = 0.60]. For the Tsik and Twitter calls, the proportion of ILD-sensitive cells was significantly smaller at lower sound levels [Tsik: χ^2^_(2)_ = 12.0, *p* = 0.002; Twitter: χ^2^_(2)_ = 13.9, *p* < 0.001], whereas ILD sensitivity did not change with level for pure tones [χ^2^_(2)_= 0.68, *p* = 0.71] or the Ock call [χ^2^_(2)_ = 2.2, *p* = 0.32].

### Sensitivity of A1 neurons to ILD–discrimination index and modulation range

**Figure 5:**
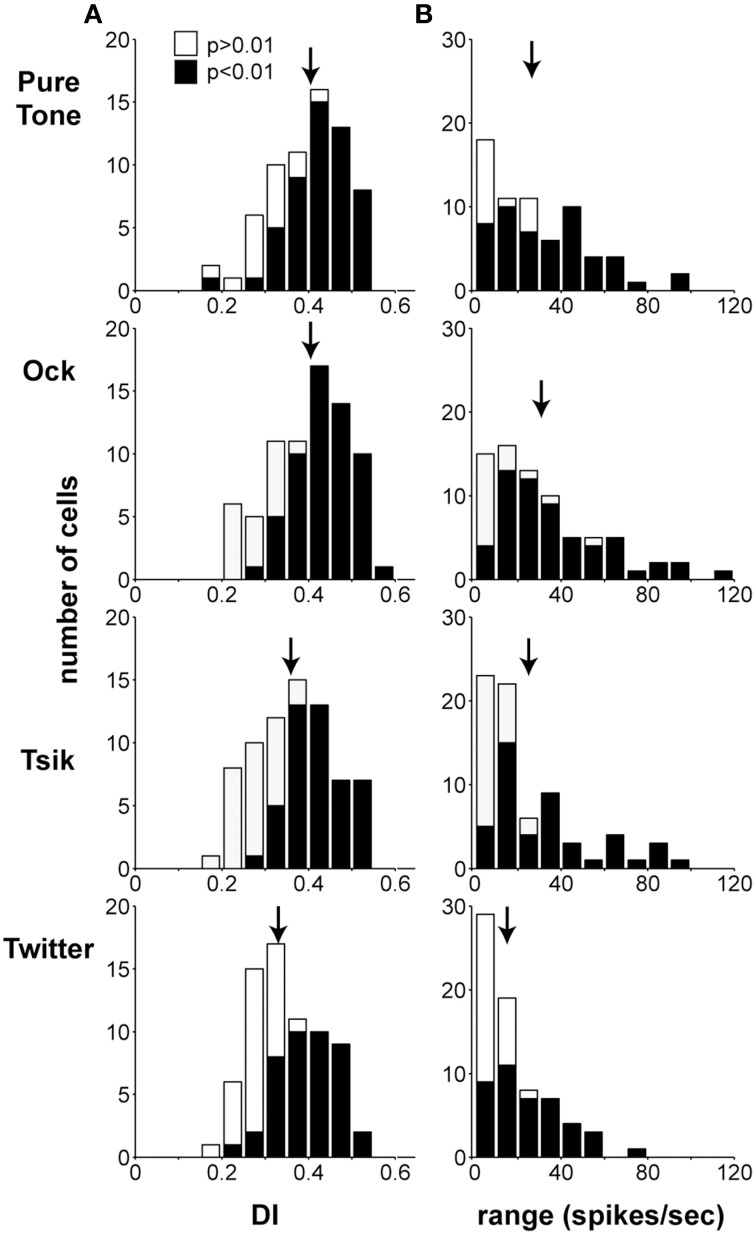
**Discrimination index (DI) and modulation range**. Column **(A)** shows the distribution of DI ABL_opt_ for all stimulus types. Column **(B)** shows the distribution of modulation range with respect to changes in ILD at the ABL_opt_. Shading of bars in both columns indicates whether or not these cells were significantly modulated in response to different ILDs. Arrows indicate the means of the distributions.

We characterized the discrimination quality afforded by the ILD sensitivity of A1 cells by calculating DIs and the modulation range of firing rates over the test ILD range. Figure 5 illustrates the distributions of DIs (Column A) and the modulation range of spike rates (Column B) for the four stimuli. The modulation range and DI were computed at each cell's optimal level (ABL_opt_), which was defined as follows: if the within-level analysis (One-Way ANOVA) revealed only one ABL with significant ILD sensitivity, that ABL was defined as ABL_opt_. Otherwise, the ABL with the highest DI was defined as ABL_opt_. Therefore, data points in each sub-panel of Figure 5 are independent, as each cell is only represented once at the ABL_opt_. Note that the DI calculation does not make any assumptions about the shape of the sensitivity function; therefore, ILD-insensitive (*p* > 0.05) cells simply have low DIs, reflecting the experimental observations. The distributions of ABI_opt_ for each stimulus are shown in Table 1; this distribution was significantly dependent on stimulus [χ^2^_(6)_ = 28.7, *p* > 0.0001]: for the pure tone, the number of cells at ABL_opt_ is relatively consistent, whereas, this number increases with ABL especially for the Tsik and Twitter calls, where more cells have ABI_opt_ of 70 dB.

The mean (±SEM) DIs for the CF tone, and the Ock, Tsik and Twitter calls were 0.3990 ± 0.0098, 0.4016 ± 0.0099, 0.3668 ± 0.0103, and 0.3483 ± 0.0094, respectively (Figure 5, Column A), significant differences were found between groups (*p* < 0.0001, repeated measures ANOVA). Note that the highest DI was found for the stimulus in our set that encompassed the largest range of frequencies (Ock), but that the next highest DI was found for the CF tones with the narrowest frequency spectrums. This however has to be interpreted with caution as the CF tones, by definition, were played at the cells' characteristic frequency, and hence the majority of cells were responsive. A similar trend was observed for the modulation range (Figure 5, Column B), with the neuronal responses to the CF pure tone (28.8 ± 2.5) and the Ock call (31.1 ± 2.8) showing higher mean modulation rates than those to the Tsik (24.3 ± 2.7) and Twitter (17.6 ± 1.7) calls; again, significant differences were evident (*p* < 0.0001 repeated measures ANOVA). *Post-hoc* tests on both measures revealed that Ock and the CF tone resulted in significantly higher DIs and modulation ranges than the Twitter call (*P* < 0.05); the remaining pair-wise comparisons did not reveal significant differences. In summary, for both CF tones and vocalizations, a large proportion of cells were modulated by ILDs. ILD sensitivity were dependent on stimulus type, ILD related modulation were strongest in response to the Ock and the CF pure tone in comparison to the Tsik and Twitter.

### Characterization of ILD sensitivity functions

For cells that were significantly ILD-sensitive (*p* < 0.005, ANOVA) we conducted analyses to characterize the sensitivity function in terms of the ILD location of the midpoint of the ILD sensitivity function and the slope of the ILD sensitivity function.

#### Lateral biases in ILD-sensitivity functions

**Figure 6:**
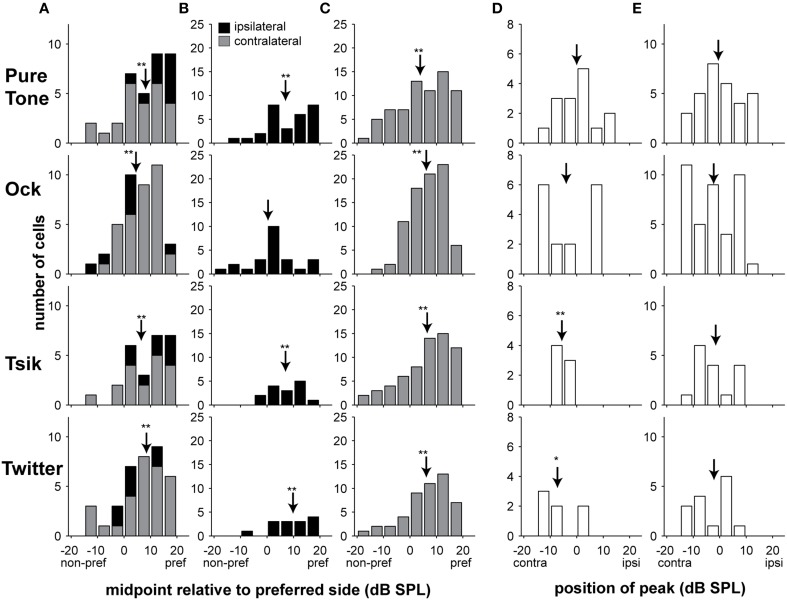
**Distribution of midpoints of ILD-response functions**. Columns **(A–C)** illustrate monotonic ILD cells for all stimulus types, the midpoints are illustrated relative to the preferred side, positive values indicate midpoints closer to the preferred side. In Column **(A)**, both ipsilateral and contralateral preferring cells are illustrated at the ABL_opt_, i.e., each data point represents the result of an individual cell. For Columns **(B–C)**, data are separated into ipsilateral and contralateral preferring groups, respectively, and all significant responses are included; in this instance, there could be up to three data points from each unit if all three intensities are significantly (monotonically) sensitive. Columns **(D,E)** show the positions of the peaks for cells that were better described by peaked ILD-response functions; positive values indicate louder in ipsilateral ear, and negative values indicate louder in the contralateral ear. In Column **(D)**, as per Column **(A)**, only results from the ABL_opt_ for peak-tuned cells are included. In Column **(E)**, all significant responses are included. Shading of bars indicates tuning type; arrows in all cases indicate the mean, ^*^ *p* < 0.05, ^**^ *p* < 0.001. Abbreviations: contra, contralateral; ipsi, ipsilateral; pref, preferred; non-pref, non-preferred.

A key parameter for characterizing the ILD sensitivity function is the midpoint, which corresponds to the point of inflection of monotonic functions (Equation 1), or the peak of Gaussian functions (Equation 2); in both cases, this is represented by parameter d_0_. For cells that are best described by a monotonic ILD-response function, this parameter indicates the point around which the neuronal responses are most sensitive to small changes in ILD. For the monotonic functions, we wanted to represent the midpoint ILD relative to the preferred side (the set of ILDs at which firing rate was maximum), independent of whether that corresponded to ILDs favoring the contralateral or ipsilateral ear. This was achieved by multiplying d_0_ by -1 for every monotonic cell that had preferred ILDs favoring the contralateral ear (parameter *A* < 0). As a result, all positive values in Figure 6, Columns A–C, indicate midpoint ILDs that are closer to the preferred set of ILDs, and conversely, negative values indicate midpoint ILDs closer to the non-preferred set of ILDs.

Figure 6 Column A shows midpoint data at only the ABL_opt_, for all cells with significant monotonic ILD sensitivity function. Each cell accounts for only one data point in the plots of Figure 6 Column A. For all stimuli, the midpoint ILDs of the monotonic sensitivity functions were generally closer to the preferred side, as indicated by the skew toward positive values in Figure 6A. These distributions were significantly different from zero (all 4 stimuli: *p* < 0.0001, one sample *t*-test; Figure 6A).

In Figure 6 Columns B and C, we separated the monotonically sensitive responses into those that preferred ILDs favoring the ipsilateral ear (B) or the contralateral ear (C). We now included in the analysis data for significant ILD-sensitive functions at non-optimal ABLs; hence in these plots, each cell could potentially account for three data points (one at each ABL). This analysis was performed separately according to laterality of the ILD sensitivity curve, given that pooling data could result in a systematic bias in favor of the more numerous type (the contralateral-preferring) of cells. The results confirm a previously observed bias in the pooled data toward the preferred ear, with only one exception: Ock ipsilateral (*p* = 0.22) where the distribution was not significantly shifted away from zero. The means of all other distributions differed significantly from zero (CF tone, ipsilateral: *p* < 0.0001, contralateral: *p* < 0.0001; Ock, contralateral: *p* < 0.0001; Tsik, ipsilateral *p* = 0.0003, contralateral: *p* < 0.0001; Twitter, ipsilateral: *p* = 0.0006, contralateral: *p* < 0.0001; one-sample *t*-test in all cases). Moreover, all distributions that were significantly different from zero had mean midpoint ILDs favoring the preferred ear, i.e., for cells that preferred the contralateral ear, the midpoints were also closer to the ILDs favoring the contralateral ear, and for cells which preferred the ipsilateral ear, the midpoints were also biased to ILDs favoring the ipsilateral ear.

We also investigated whether the position of peaks in peaked cells favored either ear. Figure 6 Column D shows data for cells with peak sensitivity at the ABL_opt_. This analysis was simply performed relative to recording hemisphere: negative values represent peak-response ILDs favoring the contralateral ear and positive values represent peak-response ILDs favoring the ipsilateral ear. The distributions of peaks for the Tsik and Twitter calls were significantly biased to ILDs favoring the contralateral ear (*p* = 0.005 and *p* = 0.04 respectively, one sample *t*-test). This effect was not seen for the Ock or CF tone stimuli (*p* > 0.05), which showed no significant bias. However, interpretation of this result must be tempered by the small number of A1 cells with peaked ILD-response for Tsik (7 units) and Twitter (7 units) calls. When we analyzed all conditions that elicited significantly peaked tuning, regardless of level (Figure 6, Column E), we also found that the distributions for all stimuli were not different from zero (*p* > 0.05). In summary, laterality effects were generally absent among the populations of cells for which ILD sensitivity was best described by peaked functions; i.e., peaked ILD functions tended to have their peaks at zero ILD which corresponds to the midline in azimuth.

#### Slope of ILD sensitivity functions

**Figure 7:**
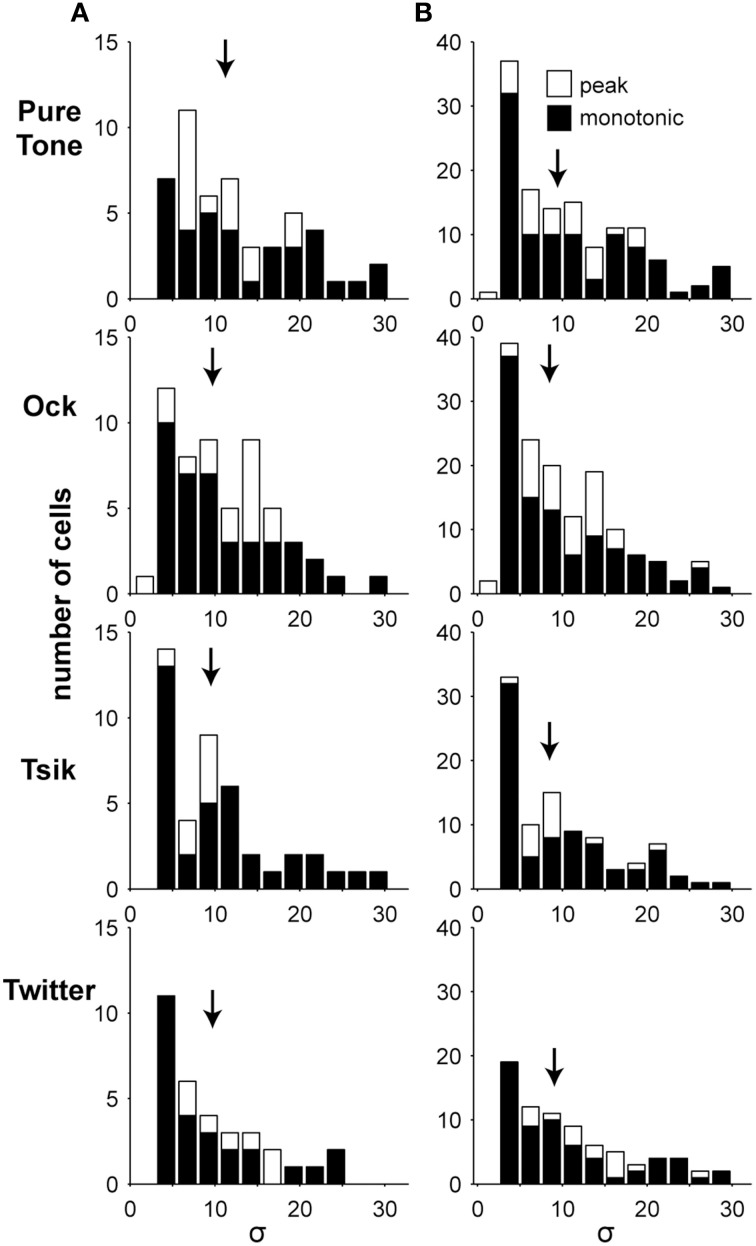
**Slope of ILD-response functions**. Each column shows the distribution of the fitted parameter σ which indicates the slope of the ILD-response functions. **(A)** In the left column, only data from the ABL_opt_ of significantly sensitive cells are illustrated, and hence there is a maximum of one data point from each cell. **(B)** In the right column, all significantly sensitive responses are shown, so there could potentially be three data points from each cell. Shading indicates the type of ILD-response function. Arrows indicate the medians of the distributions. No significant differences were found.

The slope of ILD sensitivity functions of A1 cells was quantified for both monotonic functions (Equation 1) and peaked functions (Equation 2) by the parameter σ, which has been commonly used to identify relative rates of change in sensory neuroscience for Gaussian and monotonic response functions (i.e., DeAngelis and Uka, 2003; Lui et al., 2006). Note that σ values are comparable between models/equations, with a given σ value reflecting a similar relative gradient. Indeed, when we examined the fitted σ values of both peaked and monotonically sensitive cells (at ABL_opt_; Figure 7 Column A), no difference was found between the slopes for the two types of ILD sensitivity function for any of the stimulus types (*p* > 0.05, Wilcoxon rank-sum test). Median σ values were between 10.2 and 7.2, and there was no significant difference between responses to different stimuli (*p* = 0.22, Kruskal–Wallis due to non-normal distribution; Figure 7 Column A). In real terms, the median σ values of 7.2 and 10.2 corresponded to 80% of the cell's dynamic range spanning approximately 13.1 and 18.5 dB, respectively, in ILD. When the analysis was expanded to also include responses at non-optimal ABLs (Figure 7 Column B), the same conclusion was reached (*p* > 0.05, Wilcoxon rank-sum test).

### Changes in sensitivity type with changes in ABL

Our initial analysis (Figure 4) demonstrated that the sensitivity to ILD of A1 neurons depends on stimulus level (ABL). To explore this relationship in greater detail at the level of single cell responses, we analyzed all ILD-sensitive responses as determined by Two-Way ANOVA (see Experimental Procedures). For this analysis, it was also necessary for ILD-sensitive units to be significantly sensitive to ILD at their ABL_opt_ (One-Way ANOVA); therefore, we adopted the additional criterion that the unit had to be significantly sensitive to ILD for least one of the three test ABLs in order to be considered as ILD-sensitive. With this additional criterion, several units (3 units for the pure tone, 2 for Ock, 2 for Tsik and 9 for Twitter; not necessarily the same units) were excluded from the ILD-sensitive group and not included in this analysis. Note that this discrepancy is possible because of the increase in the number of trials when data from all ABLs was considered, together with the more stringent criteria applied to the One-Way ANOVA for the reason of multiple comparisons. For cells which were significantly sensitive at only one ABL, that ABL was considered to be ABL_opt_; for units which were sensitive to ILD at multiple ABLs, the ABL that yielded the best DI was considered the ABL_opt_. Units that were not significantly selective for ILD were not included in this analysis. The total number of units with preference at each ABL for each stimulus type is shown in **Figure 10**.

**Figure 8:**
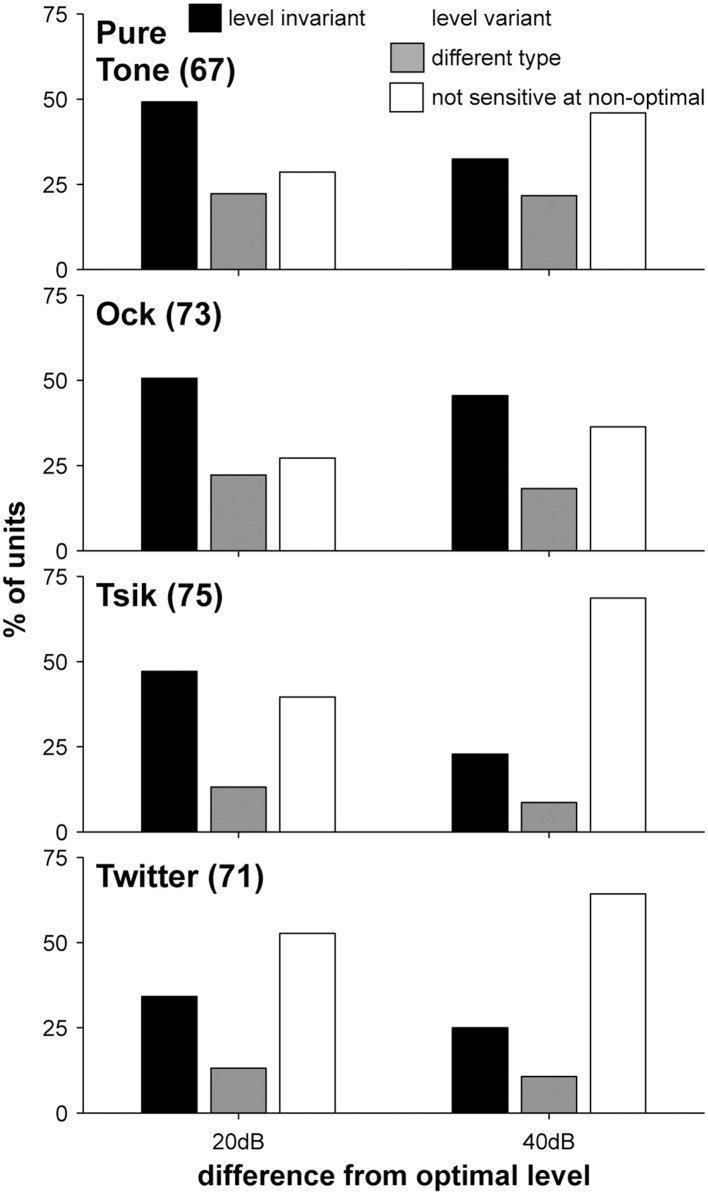
**Distribution of level invariance in ILD sensitivity with respect to stimulus type and ABL**. Sub-plots in each row illustrate data from different stimuli; left and right bar groups indicate the ABL difference from ABL_opt_. Shading indicates the percentage of each cell type as indicated by the legend. Numbers in brackets the number of cells included for each stimulus.

For a neuron to code for ILD independent of ABL, a major criterion is that it must maintain its sensitivity type (monotonic or peaked) across changes in ABL. This stability of ILD sensitivity type with changes in ABL was observed in 30–50% of cells when ABL shifted by 20–40 dB from ABL_opt_ (Figure 8). Although this is not the only criterion that needs to be fulfilled for ability to code ILD independent of ABL (as we will address below), as the first pass we classed such cells as “level invariant” sensitivity type (black bars). The remaining cells were considered to have level-variant sensitivity (gray and white bars). Of the latter group, the majority (68%) of ABL variant responses became insensitive for ILD when ABL was shifted away from optimal (white bars in Figure 8 example cell is shown in Figure 2 Cell B pure tone) while the remainder of the level-variant responses showed a change in ILD sensitivity function type when the ABL was shifted to a non-optimal level 20 dB away from ABL_opt_ (gray bars in Figure 8; example cell in Figure 2 Cell C pure tone). It could be argued that neurons with different ILD-response functions at different ABLs may still prefer the same “side” of space. However, despite such an overall preference for the same “side” of space, the different ILD sensitivity function type indicates that there will be ILDs for which these sensitivity functions may give quite different responses. In population read-outs, especially for accuracy tasks (i.e., where in space is this sound coming from?), different shapes of ILD-response functions will contribute differently to the overall read-out (see Ma et al., 2006) and this will confuse the read-out. Thus, we argue that it is important to restrict the classification of level-invariant cells only to those with the same ILD-sensitivity function, and not to broadly include event those with changes in ILD-sensitivity function but maintained preference for the same “side” of space.

**Figure 9:**
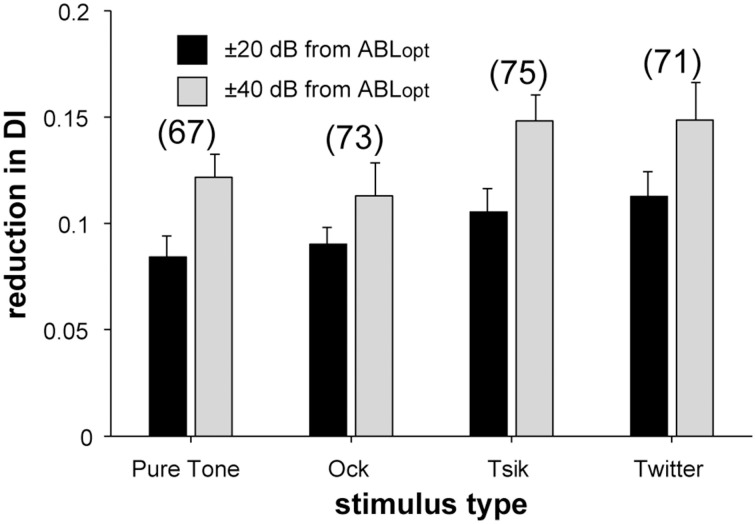
**Reduction in DI upon presentation of non-ABL_opt_ sound levels**. The difference in DI in response to ABL_opt_ and non-ABL_opt_ is illustrated for each of the four stimuli separately; bars indicate means and error bars are SEM. Color code as per the legend indicates the absolute difference of ABL from ABL_opt_. Numbers in brackets the number of cells included for each stimulus.

Level invariance depended on stimulus [χ^2^_(6)_ = 21.92, *p* = 0.0013; Figure 8]: more cells were level-invariant to the pure tone and the Ock call compared to the Tsik and Twitter calls. There was also a change in the distribution of level invariance when ABL was shifted by 20 dB from ABL_opt_ vs. when it was shifted by 40 dB from ABL_opt_ (Figure 8, left vs right). In the latter case, a smaller proportion of cells remained level-invariant [χ^2^_(2)_ = 12.8, *p* = 0.002]. These results were also reflected in the DIs: for the pure tone and Ock, the reduction in DI as ABL shifted away from ABL_opt_ was less than for Tsik or Twitter [Figure 9; main effect for stimulus type *F*_(3, 1)_ = 3.28, *p* = 0.02]. Predictably, the reduction in DI was also more substantial when the ABL was further from ABL_opt_ [Figure 9; main effect for ABL *F*_(3, 1)_ = 3.28, *p* < 0.00001]; this result was consistent for all stimuli as no interaction effect was found [*F*_(3, 1)_ = 0.28, *p* > 0.05].

In summary, 30–50% cells maintained the same sensitivity type (i.e., were level-invariant) when ABL shifted from its optimal level. The percentage of such level-invariant cells and the change in DI depended on the stimulus type and on the difference in ABL from ABL_opt_. In the following sections, we describe changes in response properties that account for these level-dependent changes.

### Level-dependent changes in ILD-response functions

**Figure 10:**
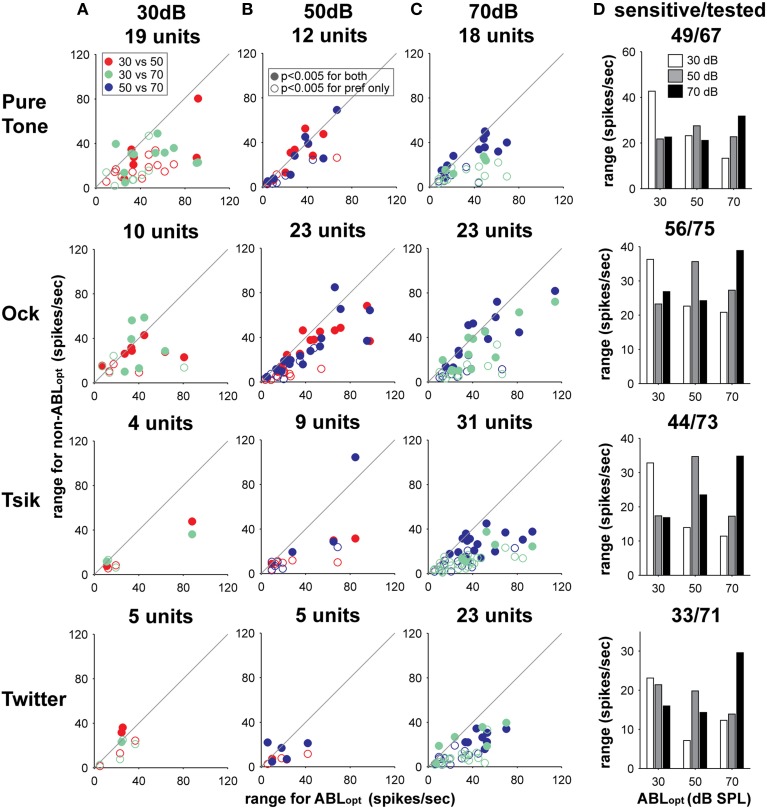
**Change in modulation range with respect to change in ABL**. Each sub-plot in columns **(A–C)** illustrates the difference in modulation range between responses to the ABL_opt_ in comparison to non-optimal intensities. Plots in columns **(A–C)** illustrate cells with different optimal intensities. Data from different call types or pure tones are shown on different rows; the identity of the stimulus is indicated on the left. The number of cells with a particular ABL_opt_ for a particular stimulus type is shown above each sub-plot in Columns **(A–C)**. The specific pair-wise comparisons for different ABLs are coded by color (see legend in Column **A**), and whether cells are significantly sensitive at the non-optimal ABL is coded according to the legend in Column **(B)**. The differences in modulation range with respect to ABL_opt_ and ABL are summarized in Column **(D)**. Also shown at the top of each sub-plot in this column are the number of cells sensitive/the total number of cells tested with the stimulus in question.

Consistent with the loss of sensitivity to ILD at non-optimal ABLs, a cell's modulation range was the most reliable indicator of the ABL_opt_ for each stimulus. Predictably, as this measure was indirectly use to calculate ABLopt, the modulation range was smaller for non-optimal ABLs in comparison to ABL_opt_ for all stimulus types (repeated measures *t*-test: *p* < 0.00001 for all four stimuli). This effect is illustrated in Columns A–C of Figure 10 which plots the modulation range for the ABL_opt_ vs. that for the non-optimal ABL. Note that for all stimuli, and for all ABL_opt_ levels (Column A: ABL_opt_ = 30 dB; Column B: ABL_opt_ = 50 dB; Column C: ABL_opt_ = 70 dB), the majority of data points fall below the line of equality, indicating lower dynamic ranges for the non-optimal ABL than the ABL_opt_ (see Figure 10. Column D for summary). As the DI which was used to calculate ABLopt is heavily dependent on the modulation range, the modulation range will be greater for ABL_opt_ in comparison to non-optimal ABLs. What is important here is that shifting the ABL from its optimal level by 20 dB will on average reduce the modulation range by approximately 40%.

**Figure 11:**
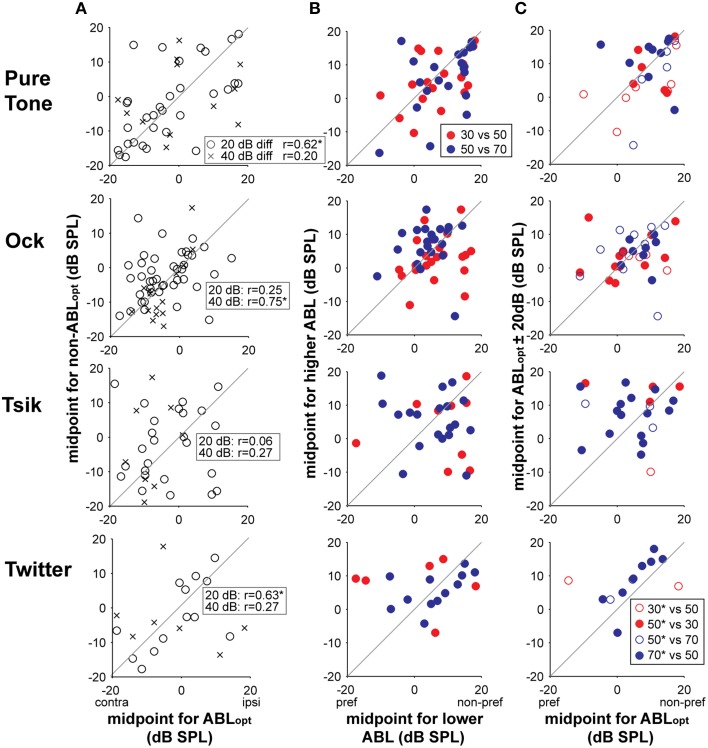
**Shifts in midpoints for ILD-response functions with respect to changes in ABL for tuning-invariant cells**. Column **(A)** compares the actual midpoints of responses for the ABL_opt_ (x-axis) and non-optimal ABLs (y-axis). The legend (top plot) indicates whether the differences are 20 or 40 dB SPL; *r*-values are indicated in each sub-plot for each difference and ^*^denotes a significant correlation (*p* < 0.05). Only level-invariant responses are shown here in these plots. Column **(B)** compares the midpoints relative to the preferred side as mean intensities are increased (regardless of the optimal). The legend denotes the specific intensities of the pair-wise comparisons. Column **(C)** compares the midpoints relative to the preferred side for optimal intensities (x-axes) with non-optimal intensities (y-axes). Only level-invariant monotonically sensitive cells are shown here. Legend identifies the specific comparisons, ^*^denotes the ABL_opt_, i.e., open circles are cells which prefer the lower ABL and closed circles are cells that prefer the higher ABL. Different rows show data in response to different stimuli. Abbreviations: contra, contralateral; ipsi, ipsilateral; pref, preferred; non-pref; non-preferred.

To determine the ability of a cell to code for ILD independent of ABL, as well as maintaining sensitivity type (as noted above), the position and rate of change of the ILD-response should also be independent of ABL. We restrict analysis to level-invariant cells since, for level-variant cells, a change in sensitivity type (including loss of tuning) already indicates that the neuron cannot code for ILD in the same way for those two ABLs; secondly, parameters fitted to a function specifically refer to specific aspects of the tuning curve for monotonic (Equation 1) and peaked (Equation 2) curves; and lastly, it is not possible to reliably compare fitted parameters when a cell has lost its sensitivity at an non-optimal ABL. The two parameters examined here, σ and d_0_, were not involved in the calculation of ABL_opt_, therefore potential differences found here can be attributed to the behavior of the neurons, and not to the method of analyses. We first evaluated the consistency of ILD-response function positions when ABL was varied, by comparing midpoints (parameter d_0_) between ILD-response functions from different ABLs (for level-invariant responses only). These data are illustrated in Column A of Figure 11. If the position of ILD-response functions was maintained across intensities, positive correlations would be evident in these data. Significant correlations were present but sporadic, being manifest at 20 dB from ABL_opt_ (circles) only for the pure tone and the Twitter call, and at 40 dB from ABL_opt_ (crosses) for the Ock call (as denoted by ^*^ beside the *r*-value in the legend). Given that the majority of the cells were sensitive to ILD in a monotonic way (see Figure 3), we tested the hypothesis that there was a systemic shift in the midpoint, either toward or away from the preferred ear, when ABL was increased by 20 dB (regardless of which ABL was optimal). While shifts in either direction were observed for individual neurons, on the population level, these shifts were not significant in one direction or the other for any of test stimulus (Figure 11 Column B; CF tone: *p* = 0.46; Ock call: *p* = 0.11; Tsik call: *p* = 0.66; Twitter call: *p* = 0.52; repeated measures *t*-test in all cases). We also tested the hypothesis that there was a systemic shift in midpoint either toward or away from the preferred ear when ABL was changed away from ABL_opt_ by 20 dB. We also did not observe any systemic changes (Figure 11 Column C; CF Tone: *p* = 0.13; Ock call: *p* = 0.21; Tsik call: *p* = 0.62; Twitter call: *p* = 0.24; repeated measures *t*-test in all cases).

**Figure 12:**
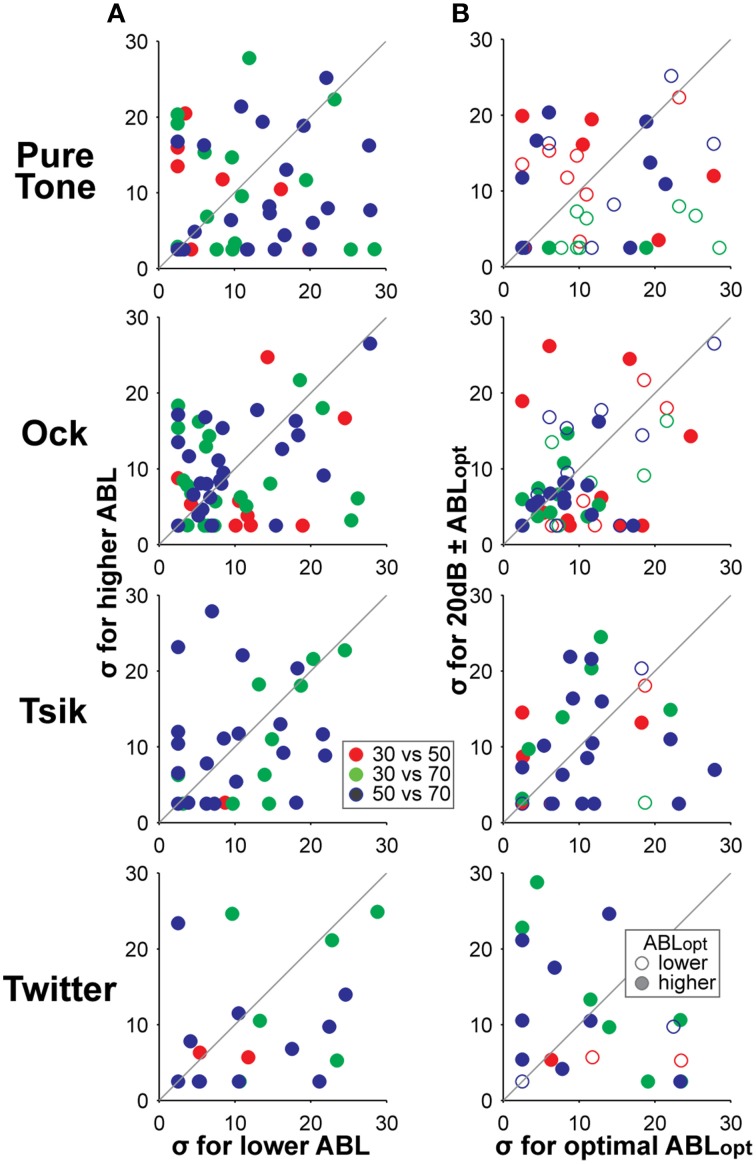
**Comparison of slopes of ILD-response functions at different ABLs**. Column **(A)** compares the slope of the ILD-response function as indicated by the fitted parameter σ, for lower (x-axis) and higher (y-axis) ABL. The ABL of the stimuli are indicated by the color coding according to the legend. Column **(B)** compares the slopes of the tuning curves when the ABL was varied from ABL_opt_ (x-axis) to one that is non-optimal (y-axis). In addition to the color code indicating stimulus ABL, we also indicate whether the ABL_opt_ of the cells was higher or lower than the comparison ABL using the legend in the bottom plot; i.e., a green filled data point indicates that the ABL_opt_ is 70 dB SPL and the comparison is made against its response to 30 dB ABL. Different rows show data in response to different stimuli. All responses shown here have tuning-invariant responses.

We also compared the rate of change in firing rates with ILD for level-invariant cells and found that the slope of the ILD-response functions did not change when stimulus ABL increased, as measured by comparing the fitted parameter σ for level-invariant responses at multiple ABLs. The results of this analysis are shown in Column A of Figure 12. While changes in slope were observed for individual cells, no significant changes with respect to increasing ABL were found for any of our stimuli when considered across the population (*p* > 0.05; Wilcoxon rank-sum test due to non-normality). We also assessed whether the slope of the ILD-response curves changed when ABL was shifted away from optimal (Figure 12 Column B). No such changes were evident across our entire stimulus set (*p* > 0.05; Wilcoxon rank-sum test).

In summary, we found that the changes in ILD-response properties with respect to ABL were mostly accounted for by the reduction in modulation range (of spike rates) when the ABL was non-optimal. The midpoints of the ILD-response functions were maintained for level-invariant cells for some, but not all, stimulus types. Finally, as a population, there were no systematic shifts in midpoints or slopes of ILD-response functions, when the ABL of the stimulus changed.

## Discussion

This study provides new information regarding ILD sensitivity of vocalization responses of A1 neurons in the marmoset, and extends previous findings of ILD tuning to CF tones. Single cell ILD responses in A1 to CF pure tones and calls were similar in that sensitivity was heavily dependent on stimulus level (ABL), and the distributions of response types (contralateral/ipsilateral monotonic or peaked) were similar. However, on a population level, more cells were responsive to the appropriate CF pure tone and to the Ock call than to the Tsik or Twitter calls, and most cells showed greater response sensitivity (e.g., greater modulation depth of responses) to the CF pure tones and the Ock call than to the Tsik or Twitter calls.

The majority of the ILD-sensitive cells had monotonic response functions with a strong bias toward the side from which stronger responses were elicited. By definition, this was obviously true of the ILDs that elicited the maximum firing rate in each cell, but we also found this bias in the midpoints of the ILD-response functions, which also tended to be displaced toward the ear eliciting the stronger responses (regardless of whether this was the contralateral or ipsilateral ear). For cells that had peaked ILD-response functions, evidence for lateralization was very weak. Regardless of the type of ILD-response function, a wide range of slopes was observed, with the dynamic range of neurons typically spanning 12–20 dB.

We also found that, for all stimuli, ILD sensitivity was heavily dependent on sound level, with a decrease in the number of neurons that exhibited the same form of ILD sensitivity when the ABL was shifted away from ABL_opt_, as well as a reduction in modulation range and DI. However, this also depended on the stimulus, and ILD-sensitive responses to the pure tone and the Ock call appear to be more robust to changes in ABL than those to the Tsik or the Twitter call. Finally, we found no systematic change in the position of the midpoints or slope of the ILD-response functions to any stimulus, with changes in stimulus level.

### Broad ILD-response functions of a1 neurons suggests a distributed population code for auditory space

Typically, spatial receptive fields in A1 span tens of degrees along the azimuth, and are confined to one hemifield or the other, with the majority of cells showing sensitivity to the contralateral hemifield (Brugge et al., 1996; Reale et al., 2003; Zhou and Wang, 2012). Our finding that most cells displayed monotonic sensitivity for ILDs that favor contralateral azimuths is consistent with this pattern, with the important caveat that ILDs do not map linearly to varying spatial locations (e.g., Martin and Webster, 1989; Middlebrooks et al., 1989; Slee and Young, 2010). While we only used the ILD cue, our results are generally compatible with those using free-field sound sources (i.e., Middlebrooks et al., 1998; Woods et al., 2006), and particularly to those in the study of Zhou and Wang (2012, 2014) which was also performed in the marmoset. At the very least, this would suggest sound localization information is available in A1 neurons upon the presentation of stimuli with ILD without ITD.

Large receptive fields that favor either hemifield may appear to conflict with behavioral results in both monkeys and humans, in which the lowest thresholds are observed near the midline (i.e., Recanzone and Beckerman, 2004). Furthermore, localization accuracy using broad-band noise and pure tones is substantially better than the width of typical A1 spatial receptive fields (Makous and Middlebrooks, 1990; Nelken et al., 2008). These findings can be reconciled by the notion of distributed coding, where a large number of broadly sensitive units combine to give information about space, rather than a point code where a small number of active neurons with small precise receptive fields for different spatial locations yield sufficient information about space, as reported in primary visual cortex (Middlebrooks et al., 1998; Stecker and Middlebrooks, 2003; Stecker et al., 2005). The concept of distributed coding of auditory space localization in A1 (e.g., Jenkins and Merzenich, 1984) is supported by the ILD-response function widths. In the marmoset, ILDs at medium to high frequencies range from approximately +25 dB to −25 dB (mainly from +20 dB to −20 dB) across the head (Slee and Young, 2010). The modulation range of a typical A1 cell in our sample covers approximately one third of this range, and therefore the call can be considered relatively broadly tuned. However, when population responses are taken into account, broad ILD responses will increase the number of cells that participate in the computation, thereby improving neural decoding by averaging out noise between more cells (see Bejjanki et al., 2011).

In distributed population codes, it has been shown both computationally (Ma et al., 2006; Law and Gold, 2009) and experimentally (Purushothaman and Bradley, 2005; Jazayeri and Movshon, 2006) that the steepest portion of the response function, if given the right situation, is the most influential with respect to perception. This portion of the ILD-response function is represented by the midpoint (parameter d_0_) in our data. For auditory spatial sensitivity, this most informative part of the response function for the majority of cells has also been shown to be located close to the midline (Stecker et al., 2005; Campbell et al., 2006). However, equal distribution of midpoints around the midline could not account for observed deficits after unilateral lesions, which are confined mostly to the contralateral space (i.e., Jenkins and Masterton, 1982; Jenkins and Merzenich, 1984; Kavanagh and Kelly, 1987; Bizley et al., 2007). We found that the midpoints of monotonic ILD-response functions were not evenly distributed around the center (0 ILD), but were shifted toward the preferred side of space. This suggests that the “center of mass” of information across the population of A1 neurons will favor the contralateral side, which could better explain the lateralization deficits observed following unilateral A1 lesions. Even so, given that (a) ILD-response functions are broad, (b) the center of mass of d_0_ is only on average 6 dB in ILD away from equality, (c) both contralateral and ipsilateral-preferring cells are present in each hemisphere, and (d) cells from both hemispheres contribute to sound localization, our results are also compatible with the fact that the best behavioral thresholds are found around the midline.

### ILD responses depend on ABL for complex naturalistic stimuli

Our results support and extend those obtained by Zhou and Wang (2012) in the marmoset, who reported changes in spatial receptive fields when the sound level of broadband noise stimuli (in their study) was varied in free-field conditions. The pattern of ILD sensitivity to CF tones we observed in marmoset A1 was similar to that described in A1 neurons from other species (e.g., Semple and Kitzes, 1993a,b; Irvine et al., 1996; Grothe et al., 2010), where ILD sensitivity was also dependent on sound level. Our data extend these findings into the domain of natural stimuli (vocalizations). By using these stimuli, we were able to demonstrate that the extent to which ILD sensitivity changes with ABL depends on the stimulus. ILD sensitivity to the pure tone or Ock call was more robust to changes in ABL than that of the Tsik or the Twitter call. Interestingly, this result correlated with the overall ILD sensitivity of the different stimuli; we stress here that this result is unlikely to be a consequence of our analysis, as we analyzed the difference in DI from that of the ABL_opt_ which would account for any differences in overall sensitivity (Figure 8). The stimulus dependent ILD sensitivity can most likely, at least in part, be attributed to the spectral composition of the stimulus relative to the cell's preference; others indeed have found that spatial responses in auditory cortex depends on stimulus bandwidth (i.e., Eisenman, 1974; Rajan et al., 1990; Clarey et al., 1995), although these studies did not use naturalistic stimuli.

Vocalizations of marmoset do not have a stereotypical template; factors like bandwidth, harmonic ratio and duration varies between animal to animal and even between calls of the same animal for a particular call type (Dimattina and Wang, 2006). We choose to use only one token per call for each experiment, as we wanted the variance in the neural responses to reflect neural noise rather than the variation in the stimulus. Moreover, while our stimuli were calls recorded from marmosets, we did not use control stimuli of comparable spectral and temporal complexity (i.e., Fukushima et al., 2014), therefore, it's difficult to conclude that these ILD responses, or differences in ILD sensitivity, can be directly attributed to the identity of these calls. The results here should be interpreted as responses to complex naturalistic stimuli of varying bandwidths and complexities.

At first sight, the proportion of ILD-sensitive neurons in our sample (~60 to ~80%; Table 1) appears less than that reported in the cat (Semple and Kitzes, 1993a), where over 90% of A1 neurons are jointly influenced by ILD and ABL. However, this may not reflect a species difference, as the majority of ILD-insensitive cells in our sample were actually unresponsive to a particular stimulus. In the Semple and Kitzes studies (1993a,b) only pure tones were used as stimuli, and only cells responsive to pure tones were then tested for ILD sensitivity. In contrast, in our study all cells were tested for ILD sensitivity. We found that 78% of cells were ILD sensitive to pure tones, and if we exclude from our pure tone tally the nine cells that were insensitive to ILDs in pure tones (but were sensitive to ILDs in at least one call), the percentage of cells sensitive to ILDs in pure tones rose to 90%. Note that we do not discount other possible factors that would explain differences between our data and those in the cat, one of which is the difference in the anesthesia regime employed in each study. The barbiturate anesthesia used by Semple and Kitzes (1993a,b) potentiates inhibition, and may have increased the amount of non-monotonic type sensitivity, a property associated with cortical inhibition (Razak and Fuzessery, 2010; see also Rajan, 1998, 2001; Rajan et al., 2013 for discussion on the effects of sufentanil).

Several studies have found that spatial receptive fields broaden as sound levels increase (Brugge et al., 1996; Middlebrooks et al., 1998; Xu et al., 1998; Reale et al., 2003; Mrsic-Flogel et al., 2005). The expansion of spatial receptive fields could be reflected in ILD-response functions in two ways: the midpoint of monotonic ILD-response functions could shift away from the preferred side toward the non-preferred side, and/or the slope of the ILD-response function (σ) could decrease. While some individual cells exhibited this behavior, a similar number of cells also exhibited the opposite, which equated to no net change for the population. This result applied for CF tones and all calls, when ABL increased. One trivial explanation for the observed pattern could be that only cells which maintained sensitivity at multiple ABLs were included in our analysis, whereas the response curves broaden when cells become insensitive at non-optimal intensities. However, other investigators have reported compatible results: while spatial receptive field sizes may change with increasing level, the positive and negative changes offset each other, which equated to no significant changes in receptive size (Mickey and Middlebrooks, 2003; Woods et al., 2006; Zhou and Wang, 2012). Although the latter studies were conducted in awake animals, we have shown that our opiate anesthetic regime yields auditory cortical recordings that are more comparable to those described for awake animals, at least in early hierarchical stages of processing such as A1 (Rajan et al., 2013). Thus, our results suggest that while ILD information is carried by different groups of cells across different intensities, neither receptive field size nor the distribution of midpoints changes with stimulus level, at least for the cell population as a whole. This pattern could serve to simplify a putative level-invariant read-out strategy.

On the population level, we also observed a disproportionate number of ILD sensitive cells which had ABL_opt_ of higher levels (Table 1), interestingly this was only observed for the calls, and was particularly pronounced for the Tsik and Twitter. Considering also that these two calls had a greater reduction in DI when ABI was shifted away from ABL_opt_, this suggests that sources of the Tsik and Twitter call can be more easily localized at higher intensities. This hypothesis, as far as we are aware, has never been tested.

### Level-invariant representation of space in A1

To understand the generation of level-invariant representations of auditory space, knowledge of the responses of single cells is critical, as population averages usually have broader sensitivity than the sum of the individual units, and neuronal read-outs leading to behavior do not necessarily follow the average of all cells. In addition to our investigation of responses to ILDs in vocalizations, we have specifically addressed the issue of the effects of shifting the sound level away from optimal on the ILD-response properties of individual neurons in A1. The principle result of such a change is that decreased ILD sensitivity is largely accounted for by modulation of spike rates; while the shape (σ) and position in ILD space (d_0_) for individual neurons may vary between ABLs, these as a population do not change systematically. These effects occurred for both vocalizations and CF pure tone stimuli.

Even for the most reliably level-invariant cells (e.g., Figure 2A, Ock), the sound level (and the stimulus type) has to be known before one can reliably infer ILD from neuronal response. Therefore, one assumes that for a system to decode space from the ILD responses of a population of A1 cells, it has to know the level of the stimulus *a priori*. Indeed, this would be the most accurate way to determine ILD: the “weights” to be given to the information carried by each neuron contributing to the overall perceptual decision can be assigned upon stimulus level. It has been suggested that neurons can change their input weights according to the reliability of evidence from different sensory channels (Fetsch et al., 2009), and a similar mechanism could function in this context. However, our data also suggest that a level-invariant read-out, in which all weights remain the same regardless of level, may be effective. For linear classifiers, adding cells that contain no information will not degrade the system's performance; thus, level-variant cells that become insensitive to ILD at non-optimal levels would not preclude level-invariant read-outs. It remains to be seen whether a large population of A1 neurons could represent ILD in an level-invariant way, either through simulation of multiple realistic single neurons (i.e., Ma et al., 2006), or via multiple neurons recorded simultaneously, which takes into account real correlations between neurons (i.e., Graf et al., 2011). Considering that we observed no population shifts in the distribution of midpoints (d_0_ parameter) and steepness of ILD-response functions with respect to ABL, in theory this increases the chances of level-invariant coding strategy being successful.

It has previously been thought that the spike rates of cortical neurons are insufficient to account for behavioral performance, with spike patterns consistently carrying more information than spike counts alone (Middlebrooks et al., 1994, 1998). More recently, it has been suggested that population spike counts of cortical neurons using an “opponent-channel” strategy can perform relatively well (Stecker et al., 2005; Miller and Recanzone, 2009). The spike counts of A1 units sampled in the present study carry more information regarding space than those investigated by Middlebrooks et al. (1994), with the positive correlation between positions of ILD-response functions (midpoints) for different levels potentially facilitating level-invariant decoding.

## Conclusions

Our study extends previous findings of ILD sensitivity of A1 neurons in response to pure tones to encompass natural marmoset vocalizations. While similar types of ILD-response functions were found for each stimulus, A1 cells were more sensitive to ILD for the Ock vocalization and the CF pure tone in comparison to the Tsik and Twitter calls. ILD sensitivity of A1 neurons was dependent on ABL; the extent which this occurred was dependent on stimulus type, reiterating that A1 responses to complex sounds cannot always be predicted by its responses to pure tones. Altogether, our results suggest that a large number of A1 neurons participate in sound localization in order to create a representation of space that's invariant of level.

### Conflict of interest statement

The authors declare that the research was conducted in the absence of any commercial or financial relationships that could be construed as a potential conflict of interest.

## References

[B1] Aitkin L. M. Merzenich M. M. Irvine D. R. Clarey J. C. Nelson J. E. (1986). Frequency representation in auditory cortex of the common marmoset (*Callithrix jacchus jacchus*). J. Comp. Neurol. 252, 175–185. 10.1002/cne.902520204 3782506

[B2] Aitkin L. Park V. (1993). Audition and the auditory pathway of a vocal New World primate, the common marmoset. Prog. Neurobiol. 41, 345–367. 10.1016/0301-0082(93)90004-C 8210411

[B3] Bejjanki V. R. Beck J. M. Lu Z. L. Pouget A. (2011). Perceptual learning as improved probabilistic inference in early sensory areas. Nat. Neurosci. 14, 642–648. 10.1038/nn.2796 21460833 PMC3329121

[B87] Bizley J. K. Nodal F. R. Parsons C. H. King A. J. (2007). Role of auditory cortex in sound localization in the midsagittal plane. J. Neurophysiology 98, 1763–1774. 10.1152/jn.00444.2007 17596417 PMC7116534

[B4] Bourne J. A. Rosa M. G. P. (2003). Preparation for the *in vivo* recording of neuronal responses in the visual cortex of anaesthetised marmosets (*Callithrix* jacchus). Brain Res. Brain Res. Protoc. 11, 168–177. 10.1016/S1385-299X(03)00044-8 12842222

[B5] Brugge J. F. Reale R. A. Hind J. E. (1996). The structure of spatial receptive fields of neurons in primary auditory cortex of the cat. J. Neurosci. 16, 4420–4437. 8699253 10.1523/JNEUROSCI.16-14-04420.1996 PMC6578856

[B6] Burman K. J. Palmer S. M. Gamberini M. Spitzer M. W. Rosa M. G. P. (2008). Anatomical and physiological definition of the motor cortex of the marmoset monkey. J. Comp. Neurol. 506, 860–876. 10.1002/cne.21580 18076083

[B7] Campbell R. A. Doubell T. P. Nodal F. R. Schnupp J. W. King A. J. (2006). Interaural timing cues do not contribute to the map of space in the ferret superior colliculus: a virtual acoustic space study. J. Neurophysiol. 95, 242–254. 10.1152/jn.00827.2005 16162823

[B9] Carrasco A. Lomber S. G. (2009). Evidence for hierarchical processing in cat auditory cortex: nonreciprocal influence of primary auditory cortex on the posterior auditory field. J. Neurosci. 29, 14323–14333. 10.1523/JNEUROSCI.2905-09.2009 19906979 PMC6665053

[B10] Clarey J. C. Barone P. Irons W. A. Samson F. K. Imig T. J. (1995). Comparison of noise and tone azimuth tuning of neurons in cat primary auditory cortex and medial geniculate body. J. Neurophysiol. 74, 961–980. 7500165 10.1152/jn.1995.74.3.961

[B11] DeAngelis G. C. Uka T. (2003). Coding of horizontal disparity and velocity by MT neurons in the alert macaque. J. Neurophysiol. 89, 1094–1111. 10.1152/jn.00717.2002 12574483

[B12] de la Mothe L. A. Blumell S. Kajikawa Y. Hackett T. A. (2006). Cortical connections of the auditory cortex in marmoset monkeys: core and medial belt regions. J. Comp. Neurol. 496, 27–71. 10.1002/cne.20923 16528722

[B13] Dimattina C. Wang X. (2006). Virtual vocalization stimuli for investigating neural representations of species-specific vocalizations. J. Neurophysiol. 95, 1244–1262. 10.1152/jn.00818.2005 16207780

[B14] Eisenman L. M. (1974). Neural encoding of sound location: an electrophysiological study in auditory cortex (AI) of the cat using free field stimuli. Brain Res. 75, 203–214. 10.1016/0006-8993(74)90742-2 4841916

[B15] Eliades S. J. Wang X. (2003). Sensory-motor interaction in the primate auditory cortex during self-initiated vocalizations. J. Neurophysiol. 89, 2194–2207. 10.1152/jn.00627.2002 12612021

[B16] Eliades S. J. Wang X. (2013). Comparison of auditory-vocal interactions across multiple types of vocalizations in marmoset auditory cortex. J. Neurophysiol. 109, 1638–1657. 10.1152/jn.00698.2012 23274315 PMC3602939

[B17] Epple G. (1968). Comparative studies on vocalization in marmoset monkeys (Hapalidae). Folia Primatol. (Basel) 8, 1–40. 10.1159/000155129 4966050

[B18] Fetsch C. R. Turner A. H. DeAngelis G. C. Angelaki D. E. (2009). Dynamic reweighting of visual and vestibular cues during self-motion perception. J. Neurosci. 29, 15601–15612. 10.1523/JNEUROSCI.2574-09.2009 20007484 PMC2824339

[B19] Fukushima M. Saunders R. C. Leopold D. A. Mishkin M. Averbeck B. B. (2014). Differential coding of conspecific vocalizations in the ventral auditory cortical stream. J. Neurosci. 34, 4665–4676. 10.1523/JNEUROSCI.3969-13.2014 24672012 PMC3965789

[B20] Gallyas F. (1979). Silver staining of myelin by means of physical development. Neurol. Res. 1, 203–209. 95356 10.1080/01616412.1979.11739553

[B21] Graf A. B. Kohn A. Jazayeri M. Movshon J. A. (2011). Decoding the activity of neuronal populations in macaque primary visual cortex. Nat. Neurosci. 14, 239–245. 10.1038/nn.2733 21217762 PMC3081541

[B22] Grothe B. Pecka M. McAlpine D. (2010). Mechanisms of sound localization in mammals. Physiol. Rev. 90, 983–1012. 10.1152/physrev.00026.2009 20664077

[B23] Heffner H. E. Heffner R. S. (1990). Effect of bilateral auditory cortex lesions on sound localization in Japanese macaques. J. Neurophysiol. 64, 915–931. 2230934 10.1152/jn.1990.64.3.915

[B24] Irvine D. R. (1987). A comparison of two methods for the measurement of neural sensitivity to interaural intensity differences. Hear. Res. 30, 169–179. 10.1016/0378-5955(87)90134-1 2824421

[B25] Irvine D. R. Rajan R. Aitkin L. M. (1996). Sensitivity to interaural intensity differences of neurons in primary auditory cortex of the cat. I. types of sensitivity and effects of variations in sound pressure level. J. Neurophysiol. 75, 75–96. 8822543 10.1152/jn.1996.75.1.75

[B26] Jazayeri M. Movshon J. A. (2006). Optimal representation of sensory information by neural populations. Nat. Neurosci. 9, 690–696. 10.1038/nn1691 16617339

[B27] Jenkins W. M. Masterton R. B. (1982). Sound localization: effects of unilateral lesions in central auditory system. J. Neurophysiol. 47, 987–1016. 7108581 10.1152/jn.1982.47.6.987

[B28] Jenkins W. M. Merzenich M. M. (1984). Role of cat primary auditory cortex for sound-localization behavior. J. Neurophysiol. 52, 819–847. 6512590 10.1152/jn.1984.52.5.819

[B29] Jin C. Corderoy A. Carlile S. Van Schaik A. (2004). Contrasting monaural and interaural spectral cues for human sound localization. J. Acoust. Soc. Am. 115, 3124–3141. 10.1121/1.1736649 15237837

[B30] Kavanagh G. L. Kelly J. B. (1987). Contribution of auditory cortex to sound localization by the ferret (*Mustela putorius*). J. Neurophysiol. 57, 1746–1766. 3598629 10.1152/jn.1987.57.6.1746

[B85] Kusmierek P. Rauscheker J. P. (2014). Selectivity for space and time in early areas of the auditory dorsal stream in the rhesus monkey. J. Neurophysiology 111, 1671–1685. 10.1152/jn.00436.2013 24501260 PMC4035775

[B31] Law C. T. Gold J. I. (2009). Reinforcement learning can account for associative and perceptual learning on a visual-decision task. Nat. Neurosci. 12, 655–663. 10.1038/nn.2304 19377473 PMC2674144

[B34] Lu T. Liang L. Wang X. (2001). Temporal and rate representations of time-varying signals in the auditory cortex of awake primates. Nat. Neurosci. 4, 1131–1138. 10.1038/nn737 11593234

[B35] Lui L. L. Bourne J. A. Rosa M. G. (2006). Functional response properties of neurons in the dorsomedial visual area of New World monkeys (*Callithrix jacchus*). Cereb. Cortex 16, 162–177. 10.1093/cercor/bhi094 15858163

[B36] Ma W. J. Beck J. M. Latham P. E. Pouget A. (2006). Bayesian inference with probabilistic population codes. Nat. Neurosci. 9, 1432–1438. 10.1038/nn1790 17057707

[B37] Makous J. C. Middlebrooks J. C. (1990). Two-dimensional sound localization by human listeners. J. Acoust. Soc. Am. 87, 2188–2200. 10.1121/1.399186 2348023

[B38] Martin R. L. Webster W. R. (1989). Interaural sound pressure level differences associated with sound-source locations in the frontal hemifield of the domestic cat. Hear. Res. 38, 289–302. 10.1016/0378-5955(89)90072-5 2708168

[B39] Mickey B. J. Middlebrooks J. C. (2003). Representation of auditory space by cortical neurons in awake cats. J. Neurosci. 23, 8649–8663. 14507964 10.1523/JNEUROSCI.23-25-08649.2003 PMC6740412

[B40] Middlebrooks J. C. Clock A. E. Xu L. Green D. M. (1994). A panoramic code for sound location by cortical neurons. Science 264, 842–844. 10.1126/science.8171339 8171339

[B41] Middlebrooks J. C. Makous J. C. Green D. M. (1989). Directional sensitivity of sound-pressure levels in the human ear canal. J. Acoust. Soc. Am. 86, 89–108. 10.1121/1.398224 2754111

[B42] Middlebrooks J. C. Xu L. Eddins A. C. Green D. M. (1998). Codes for sound-source location in nontonotopic auditory cortex. J. Neurophysiol. 80, 863–881. 9705474 10.1152/jn.1998.80.2.863

[B43] Miller C. T. Beck K. Meade B. Wang X. (2009). Antiphonal call timing in marmosets is behaviorally significant: interactive playback experiments. J. Comp. Physiol. A Neuroethol. Sens. Neural Behav. Physiol. 195, 783–789. 10.1007/s00359-009-0456-1 19597736 PMC3787898

[B44] Miller L. M. Recanzone G. H. (2009). Populations of auditory cortical neurons can accurately encode acoustic space across stimulus intensity. Proc. Natl. Acad. Sci. U.S.A. 106, 5931–5935. 10.1073/pnas.0901023106 19321750 PMC2667094

[B45] Mrsic-Flogel T. D. King A. J. Schnupp J. W. (2005). Encoding of virtual acoustic space stimuli by neurons in ferret primary auditory cortex. J. Neurophysiol. 93, 3489–3503. 10.1152/jn.00748.2004 15659534

[B86] Nagarajan S. S. Cheung S. W. Bedenbaugh P. Beitel R. E. Schreiner C. E. Merzenich M. M. (2002). Representation of spectral and temporal envelope of twitter vocalizations in common marmoset primary auditory cortex. J. Neurophysiology 87, 1723–1737. 10.1152/jn.00632.2001 11929894

[B84] Nelken I. Bizley J. K. Nodal F. R. Ahmed B. King A. J. Schnupp J. W. (2008). Responses of auditory cortex to complex stimuli: functional organization revealed using intrinsic optical signals. J. Neurophysiology 99, 1928–1941. 10.1152/jn.00469.2007 18272880 PMC7116535

[B47] Paxinos G. Watson C. Petrides M. Rosa M. G. P. Tokuno H. (2012). The Marmoset Brain in Stereotaxic Coordinates. San Diego, CA: Academic Press.

[B48] Phillips D. P. Irvine D. R. (1981). Responses of single neurons in physiologically defined area AI of cat cerebral cortex: sensitivity to interaural intensity differences. Hear. Res. 4, 299–307. 10.1016/0378-5955(81)90014-9 7263517

[B49] Phillips D. P. Irvine D. R. (1983). Some features of binaural input to single neurons in physiologically defined area AI of cat cerebral cortex. J. Neurophysiol. 49, 383–395. 6834083 10.1152/jn.1983.49.2.383

[B50] Prince S. J. Pointon A. D. Cumming B. G. Parker A. J. (2002). Quantitative analysis of the responses of V1 neurons to horizontal disparity in dynamic random-dot stereograms. J. Neurophysiol. 87, 191–208. 11784742 10.1152/jn.00465.2000

[B51] Purushothaman G. Bradley D. C. (2005). Neural population code for fine perceptual decisions in area MT. Nat. Neurosci. 8, 99–106. 10.1038/nn1373 15608633

[B52] Rajan R. (1998). Receptor organ damage causes loss of cortical surround inhibition without topographic map plasticity. Nat. Neurosci. 1, 138–143. 10.1038/388 10195129

[B53] Rajan R. (2000). Centrifugal pathways protect hearing sensitivity at the cochlea in noisy environments that exacerbate the damage induced by loud sound. J. Neurosci. 20, 6684–6693. 10964973 10.1523/JNEUROSCI.20-17-06684.2000 PMC6772970

[B54] Rajan R. (2001). Plasticity of excitation and inhibition in the receptive field of primary auditory cortical neurons after limited receptor organ damage. Cereb. Cortex 11, 171–182. 10.1093/cercor/11.2.171 11208672

[B55] Rajan R. Aitkin L. M. Irvine D. R. McKay J. (1990). Azimuthal sensitivity of neurons in primary auditory cortex of cats. I. Types of sensitivity and the effects of variations in stimulus parameters. J. Neurophysiol. 64, 872–887. 2230931 10.1152/jn.1990.64.3.872

[B56] Rajan R. Dubaj V. Reser D. H. Rosa M. G. P. (2013). Auditory cortex of the marmoset monkey - complex responses to tones and vocalizations under opiate anaesthesia in core and belt areas. Eur. J. Neurosci. 37, 924–941. 10.1111/ejn.12092 23278961

[B57] Razak K. A. Fuzessery Z. M. (2010). GABA shapes a systematic map of binaural sensitivity in the auditory cortex. J. Neurophysiol. 104, 517–528. 10.1152/jn.00294.2010 20484524 PMC2904205

[B58] Reale R. A. Jenison R. L. Brugge J. F. (2003). Directional sensitivity of neurons in the primary auditory (AI) cortex: effects of sound-source intensity level. J. Neurophysiol. 89, 1024–1038. 10.1152/jn.00563.2002 12574478

[B59] Recanzone G. H. Beckerman N. S. (2004). Effects of intensity and location on sound location discrimination in macaque monkeys. Hear. Res. 198, 116–124. 10.1016/j.heares.2004.07.017 15567608

[B60] Recanzone G. H. Guard D. C. Phan M. L. Su T. K. (2000). Correlation between the activity of single auditory cortical neurons and sound-localization behavior in the macaque monkey. J Neurophysiol 83, 2723–2739. 10805672 10.1152/jn.2000.83.5.2723

[B61] Reser D. H. Burman K. J. Richardson K. E. Spitzer M. W. Rosa M. G. P. (2009). Connections of the marmoset rostrotemporal auditory area: express pathways for analysis of affective content in hearing. Eur. J. Neurosci. 30, 578–592. 10.1111/j.1460-9568.2009.06846.x 19663937

[B63] Sabin A. T. Macpherson E. A. Middlebrooks J. C. (2005). Human sound localization at near-threshold levels. Hear. Res. 199, 124–134. 10.1016/j.heares.2004.08.001 15574307

[B64] Sadagopan S. Wang X. (2009). Nonlinear spectrotemporal interactions underlying selectivity for complex sounds in auditory cortex. J. Neurosci. 29, 11192–11202. 10.1523/JNEUROSCI.1286-09.2009 19741126 PMC2757444

[B65] Semple M. N. Kitzes L. M. (1993a). Binaural processing of sound pressure level in cat primary auditory cortex: evidence for a representation based on absolute levels rather than interaural level differences. J. Neurophysiol. 69, 449–461. 8459277 10.1152/jn.1993.69.2.449

[B66] Semple M. N. Kitzes L. M. (1993b). Focal selectivity for binaural sound pressure level in cat primary auditory cortex: two-way intensity network tuning. J. Neurophysiol. 69, 462–473. 8459278 10.1152/jn.1993.69.2.462

[B67] Slee S. J. Young E. D. (2010). Sound localization cues in the marmoset monkey. Hear. Res. 260, 96–108. 10.1016/j.heares.2009.12.001 19963054 PMC2819082

[B68] Slee S. J. Young E. D. (2013). Linear processing of interaural level difference underlies spatial tuning in the nucleus of the brachium of the inferior colliculus. J. Neurosci. 33, 3891–3904. 10.1523/JNEUROSCI.3437-12.2013 23447600 PMC3613225

[B69] Stecker G. C. Harrington I. A. Middlebrooks J. C. (2005). Location coding by opponent neural populations in the auditory cortex. PLoS Biol. 3:e78. 10.1371/journal.pbio.0030078 15736980 PMC1044834

[B70] Stecker G. C. Middlebrooks J. C. (2003). Distributed coding of sound locations in the auditory cortex. Biol. Cybern. 89, 341–349. 10.1007/s00422-003-0439-1 14669014

[B71] Stevenson M. F. Poole T. B. (1976). An ethogram of the common marmoset (*Calithrix jacchus jacchus*): general behavioural repertoire. Anim. Behav. 24, 428–451. 10.1016/S0003-3472(76)80053-X 820223

[B72] Su T. I. Recanzone G. H. (2001). Differential effect of near-threshold stimulus intensities on sound localization performance in azimuth and elevation in normal human subjects. J. Assoc. Res. Otolaryngol. 2, 246–256. 10.1007/s101620010073 11669397 PMC3201671

[B73] Thompson G. C. Cortez A. M. (1983). The inability of squirrel monkeys to localize sound after unilateral ablation of auditory cortex. Behav. Brain Res. 8, 211–216. 10.1016/0166-4328(83)90055-4 6860463

[B74] Vliegen J. Van Opstal A. J. (2004). The influence of duration and level on human sound localization. J. Acoust. Soc. Am. 115, 1705–1713. 10.1121/1.1687423 15101649

[B75] Wang X. (2000). On cortical coding of vocal communication sounds in primates. Proc. Natl. Acad. Sci. U.S.A. 97, 11843–11849. 10.1073/pnas.97.22.11843 11050218 PMC34358

[B76] Wang X. Merzenich M. M. Beitel R. Schreiner C. E. (1995). Representation of a species-specific vocalization in the primary auditory cortex of the common marmoset: temporal and spectral characteristics. J. Neurophysiol. 74, 2685–2706. 8747224 10.1152/jn.1995.74.6.2685

[B77] Wightman F. L. Kistler D. J. (1997). Monaural sound localization revisited. J. Acoust. Soc. Am. 101, 1050–1063. 10.1121/1.418029 9035397

[B78] Wise L. Z. Irvine D. R. (1983). Auditory response properties of neurons in deep layers of cat superior colliculus. J. Neurophysiol. 49, 674–685. 6834093 10.1152/jn.1983.49.3.674

[B79] Wong-Riley M. (1979). Changes in the visual system of monocularly sutured or enucleated cats demonstrable with cytochrome oxidase histochemistry. Brain Res. 171, 11–28. 10.1016/0006-8993(79)90728-5 223730

[B80] Woods T. M. Lopez S. E. Long J. H. Rahman J. E. Recanzone G. H. (2006). Effects of stimulus azimuth and intensity on the single-neuron activity in the auditory cortex of the alert macaque monkey. J. Neurophysiol. 96, 3323–3337. 10.1152/jn.00392.2006 16943318

[B81] Xu L. Furukawa S. Middlebrooks J. C. (1998). Sensitivity to sound-source elevation in nontonotopic auditory cortex. J. Neurophysiol. 80, 882–894. 9705475 10.1152/jn.1998.80.2.882

[B82] Zhou Y. Wang X. (2012). Level dependence of spatial processing in the primate auditory cortex. J. Neurophysiol. 108, 810–826. 10.1152/jn.00500.2011 22592309 PMC3424089

[B83] Zhou Y. Wang X. (2014). Spatially extended forward suppression in primate auditory cortex. Eur. J. Neurosci. 39, 919–933. 10.1111/ejn.12460 24372934

